# The impact of traditional mind–body exercises on pulmonary function, exercise capacity, and quality of life in patients with lung cancer: a systematic review and meta-analysis

**DOI:** 10.3389/fonc.2025.1716583

**Published:** 2025-12-09

**Authors:** Mingqi Wu, Sidi Zhang, Hongka Zhang, Yijun Yin, Shiyun Wang, Weiwei Li, Jing Xiao, Zhanying Tang

**Affiliations:** Longhua Hospital Affiliated to Shanghai University of Traditional Chinese Medicine, Shanghai, China

**Keywords:** Qigong, Taichi, Baduanjin, Liuzijue, Wuqinx, lung cancer, meta-analysis

## Abstract

**Background:**

A systematic evaluation of the clinical effects of mind-body traditional Chinese exercises (Qigong, Taichi, Baduanjin, Liuzijue and Wuqinxi) lung cancer patients.

**Methods:**

Systematic searches of PubMed, Embase, Cochrane Library, Web of Science and China Knowledge Network (CNKI), Wan fang, China Biology Medicine disc (CBM), and VIP were performed from the time of library construction to August 20, 2025. Lung cancer patients were included in the study, and the interventions were Qigong, Taichi, Baduanjin, Liuzijue or Wuqinxi, and the control group was conventional treatment or other non-exercise interventions. Meta-analysis was performed using Stata 15.

**Results:**

22 randomized controlled studies involving 1827 patients including, meta-analysis results suggested that mind-body traditional Chinese exercises were able to reduce anxiety scores [SMD=-1.05, 95%CI (- 1.34, -0.76)], depression scores [SMD=-1.05, 95%CI (- 1.26, -0.82)], PSQI [SMD=-0.67, 95%CI (-1.17, -0.17)] and increase 6MWT [WMD = 31.24, 95%CI (19.54, 42.95)], quality of life scores [SMD = 0.85, 95%CI (0.36, 1.34)], FEV1 [WMD = 0.14, 95%CI (0.06, 0.23)],FVC [WMD = 0.08, 95%CI (0.02, 0.15)] in lung cancer patients.

**Conclusion:**

In this study, the results showed that the mind-body traditional Chinese exercises maybe significantly improve the patients’ anxiety, depression, sleep quality, exercise capacity, lung function and quality of life.

**Systematic review registration:**

https://www.crd.york.ac.uk/prospero/, identifier CRD420251127148.

## Introduction

Lung cancer ranks among the most common malignant tumors worldwide, with persistently high incidence and mortality rates (1). According to the Global Cancer Statistics 2020 report, lung cancer accounts for approximately 11.4% of all new cancer cases and causes 18% of cancer-related deaths (2). In China, lung cancer has become the deadliest form of cancer, accounting for over 25% of all cancer deaths (3). Despite advances in medical technology in recent years, which have led to some progress in early diagnosis and treatment, the high incidence rate and low early screening rate mean most patients are still diagnosed at an advanced stage (4, 5). This results in poor treatment outcomes and shorter survival times. Therefore, identifying effective adjuvant therapies has become crucial for improving treatment efficacy and quality of life for lung cancer patients (6).

Current standard treatments for lung cancer include surgery, radiotherapy, chemotherapy, targeted therapy, and immunotherapy. While these approaches have improved survival rates to some extent, their effectiveness in enhancing patients’ quality of life and alleviating treatment-related symptoms—such as cancer-related fatigue, dyspnea, and psychological stress—remains limited (7). Cancer-related fatigue is a common symptom among lung cancer patients, affecting not only physical strength but also emotional well-being, social interactions, and daily activities, significantly diminishing quality of life (8). Concurrently, patients often experience psychological issues like depression and anxiety, which traditional treatments struggle to comprehensively address (9). Consequently, identifying adjunctive therapies that simultaneously improve both physical capacity and mental health has become particularly crucial. Research suggests that exercise can enhance cardiovascular health, muscle strength, and mental well-being, but may also lead to injuries or overtraining. Traditional Chinese exercises such as Tai Chi and Qigong, on the other hand, improve flexibility and relaxation by balancing mind and body, though they may yield slower gains in strength and cardiovascular endurance (10).

Against this backdrop, traditional Chinese medicine (TCM) therapies have gradually entered the realm of adjunctive treatment for lung cancer patients (11). These therapies emphasize promoting the body’s self-repair and overall health by regulating qi and blood, internal organs, and mental states (12). Beyond physical conditioning, they stress achieving mind-body integration through coordinated breathing, mental focus, and movement. Qigong, Tai Chi, Baduanjin, Wuqinxi, and the Liuzijue represent some of the more typical forms within TCM exercise therapy, which have seen increasing application in cancer rehabilitation in recent years (13).

Qigong is a traditional exercise form that promotes health through breath regulation, slow movements, and focused intention. Its core principle is “regulating qi for health preservation” (14), enhancing immunity, improving blood circulation, and alleviating stress by harmonizing the flow of qi (15). Tai Chi, a practice integrating philosophy, martial arts, and wellness, emphasizes the harmony of stillness and movement through slow, gentle motions and deep breathing to regulate energy flow (16). It improves physical function, enhances balance, and promotes mental relaxation (17).

Baduanjin and Wuqinxi are two traditional Chinese qigong exercises originating from ancient China. They enhance physical constitution by mimicking animal movements (such as tigers, deer, bears, apes, and birds) and coordinating breath with movement, thereby promoting qi and blood circulation for health benefits (18). Baduanjin features simple, easy-to-learn movements suitable for all populations, particularly lung cancer patients undergoing rehabilitation training (19). Wuqinxi, meanwhile, engages distinct muscle groups by mimicking the behaviors of five animals, offering strong elements of fun and interactivity (20).

As another unique TCM fitness method, the Liuzijue regulates the respiratory system through six vocalized movements—”Xu, Hu, He, Si, Chui, Xi” to regulate the respiratory system, enhance qi and blood circulation, and promote physical and mental well-being (21). The Liuzijue features simple, easy-to-master movements emphasizing the coordination of deep breathing with vocalization. This practice not only relaxes muscles and alleviates stress but also improves respiratory function (22). Recent studies have found that applying the Liuzijue to lung cancer patients effectively relieves fatigue, enhances lung function, and improves psychological well-being to a certain extent (23). Unlike qigong and tai chi, the Liuzijue distinguishes itself through its unique integration of vocalization and breathing, delivering significant respiratory training benefits for the lungs. It is particularly suitable for lung cancer patients requiring increased lung capacity and improved breath control.

Although numerous studies (19, 24) indicate that traditional Chinese exercises such as qigong, tai chi, Baduanjin, Wuqinxi, and the Liuzijue positively impact lung cancer patient recovery, existing research findings often exhibit heterogeneity. Substantial variations exist across studies in intervention intensity, duration, and assessment metrics, limiting the comparability of findings (25). Moreover, most existing literature consists of small-sample, single-center studies, lacking high-quality, large-scale randomized controlled trials, which limits a comprehensive assessment of the efficacy of these exercise therapies.

Therefore, this study aims to integrate existing research on the effects of qigong, tai chi, Baduanjin, Wuqinxi, and Liuzijue on lung cancer patients through systematic review and meta-analysis. It seeks to comprehensively evaluate their clinical efficacy in improving quality of life, alleviating cancer-related fatigue, enhancing lung function, and promoting mental health among lung cancer patients. This study aims to provide additional evidence-based medical support for the comprehensive treatment of lung cancer patients and promote the application of traditional Chinese medicine exercise therapy in lung cancer rehabilitation. By incorporating more Chinese databases and employing subgroup analysis, we explore the efficacy of different traditional mind–body exercises.

## Methods

This systematic evaluation and meta-analysis will strictly follow the PRISMA (Preferred Reporting Items for Systematic Reviews and Meta-Analyses) guidelines (26). And it is registered in Prospero with registration number CRD420251127148.

### Inclusion and exclusion criteria

#### Inclusion criteria

1. The study was conducted in adult patients with lung cancer.
2. The intervention consisted of any one or more of the traditional Chinese physical and mental exercises, such as Qigong, Taichi, Baduanjin, Liuzijue and Wuqinxi. All interventions must be performed by professionally trained instructors or coaches to ensure the standardization of exercises and patient safety.
3. The control group is the conventional treatment group or other non-exercise intervention group. Conventional treatment includes standard lung cancer treatments such as surgery, chemotherapy, and radiotherapy.
4. The primary outcomes of this study included Anxiety [Hamilton Anxiety Rating Scale (HARS)], Depression [Beck Depression Inventory (BDI)], PSQI (Pittsburgh Sleep Quality Index), 6MWT (6-minute walk test), FEV1 (Forced Expiratory Volume in 1 second), and FVC (Forced Vital Capacity). These instruments have been widely validated in clinical populations, including patients with lung cancer.
5. Included studies had to be randomized controlled trials (RCTs).

#### Exclusion criteria

1. The study is designed for patients who are not lung cancer patients or who have other serious diseases (advanced heart disease, liver and kidney failure, stroke) and these diseases may interfere with the effectiveness of the intervention.
2. Patients received treatment or interventions for complications other than standard treatment during the study period that may have affected the outcome of the study.
3. The study does not clearly describe the specifics, methodology, or duration of the intervention, or the intervention does not meet the inclusion criteria for this study.
4. The study design was a non-randomized controlled trial or lacked sufficient control group data.
5. The study lacked valid outcome data or had incomplete outcome data for appropriate Meta-analysis. The study is non-publicly published gray literature or the results of the study are not available through public sources.

### Literature retrieval

Two researchers conducted independent systematic searches of the following databases: PubMed, Embase, Cochrane Library, Web of Science and China Knowledge Network (CNKI), Wan fang, China Biology Medicine disc (CBM) and VIP were performed from the time of library construction to August 20, 2025. The search strategy combined Medical Subject Headings (MeSH) with free-text terms, consisting of three main components: “Qigong[Title/Abstract]”, “Taichi[Title/Abstract]”, “Baduanjin[Title/Abstract]”, “Liuzijue[Title/Abstract]”, “Wuqinx[Title/Abstract]” and “lung cancer[Title/Abstract]”. The specific search strategy is detailed in **Supplementary Material Table S1**. The search strategy was adjusted appropriately for each database based on its characteristics. To further ensure the comprehensiveness of the literature, this study also manually searched the reference lists of included studies to supplement any potentially overlooked relevant research. In cases of disagreement during the search and screening process, a third researcher was involved to mediate and resolve the issue.

### Data extractions

This study was conducted by two researchers who independently extracted relevant data from the eligible literature using an Excel sheet based on the inclusion criteria. The extraction included the basic information of the study (first author, year of publication), the basic characteristics of the study population (sample size, gender and mean age), intervention and outcome. In the process of data extraction, if two investigators disagreed on the data, it would be resolved through negotiation, and if no agreement could be reached, a third investigator would adjudicate to ensure the accuracy and consistency of data extraction.

### Outcomes

#### Anxiety score

Hamilton Anxiety Rating Scale (HARS), This scale ranges from 0 to 56, with higher scores indicating more severe anxiety. A score of 0–17 is considered mild anxiety, 18–24 moderate anxiety, and 25–56 severe anxiety. A higher score corresponds to worse anxiety (27).

#### Depression score

The BDI has a total score range of 0 to 63, with higher scores indicating more severe depression. Scores are interpreted as follows: 0-13 (minimal depression), 14-19 (mild depression), 20-28 (moderate depression), and 29-63 (severe depression). A higher score represents worse depressive symptoms (28).

#### PSQI

The PSQI ranges from 0 to 21, with higher scores indicating poorer sleep quality. A score of 5 or greater suggests poor sleep quality. Higher scores represent worse sleep quality (29).

#### 6MWT

The 6MWT measures the distance walked in six minutes. Higher scores indicate better functional capacity and physical endurance. In patients with lung cancer, a shorter distance walked correlates with worse physical health (30).

FEV1 and FVC: Both are measured in liters, with higher values indicating better lung function. A lower FEV1 or FVC value indicates worse pulmonary function. These measures are critical in assessing respiratory health, particularly in lung cancer patients (31).

The secondary outcome was Quality of Life, measured using the SF-36 (Short Form Health Survey), which has been extensively validated across various populations, including cancer patients, and provides a comprehensive measure of health-related quality of life. The SF-36 scores range from 0 to 100, with higher scores indicating better quality of life (32).

### Risk of bias

Risk of bias was assessed by following the latest recommendations of the Cochrane Handbook Risk of Bias assessment tool 2.0 (ROB 2.0) (33), consisting of five main sections: bias arising from randomization, bias from deviations from established interventions, bias from missing outcome data, bias from outcome measures and outcome selective reporting bias. Studies were rated as “Low risk of bias”, “Some concerns”, “High risk of bias “. Two investigators independently assessed the risk of bias, and if there was disagreement, consensus was discussed, or a third party was consulted.

### Data analysis

The data analysis section utilized Stata 15.0 software (Stata Corp, College Station, TX, USA) for statistical analysis. In this study, data from the included studies were analyzed using post-intervention values. This approach was chosen to assess the effect of the intervention after the treatment period, ensuring that the meta-analysis reflects the outcomes directly resulting from the intervention. First, heterogeneity among the included studies was assessed using the *I²* value or Q statistic. *I²* values of 0%, 25%, 50%, and 75% indicate no heterogeneity, low heterogeneity, moderate heterogeneity, and high heterogeneity, respectively. When the *I²* value reached or exceeded 50%, sensitivity analysis was performed to explore potential sources of heterogeneity. If heterogeneity was below 50%, a fixed-effects model was used for analysis. For continuous variables, the standardized mean difference (SMD) and its 95% confidence interval (CI) are used; but for 6MWT, FEV1, FVC the weighted mean difference (WMD) and its 95% confidence interval (CI) are used; for dichotomous variables, the risk ratio (RR) and its 95% confidence interval (CI) are used. Additionally, a random-effects model and Egger’s test are used to assess publication bias. If the funnel plot is asymmetric, trim-and-trim methods are used for assessment to correct for potential publication bias.

## Results

### Literature search screening results

**Figure 1:**
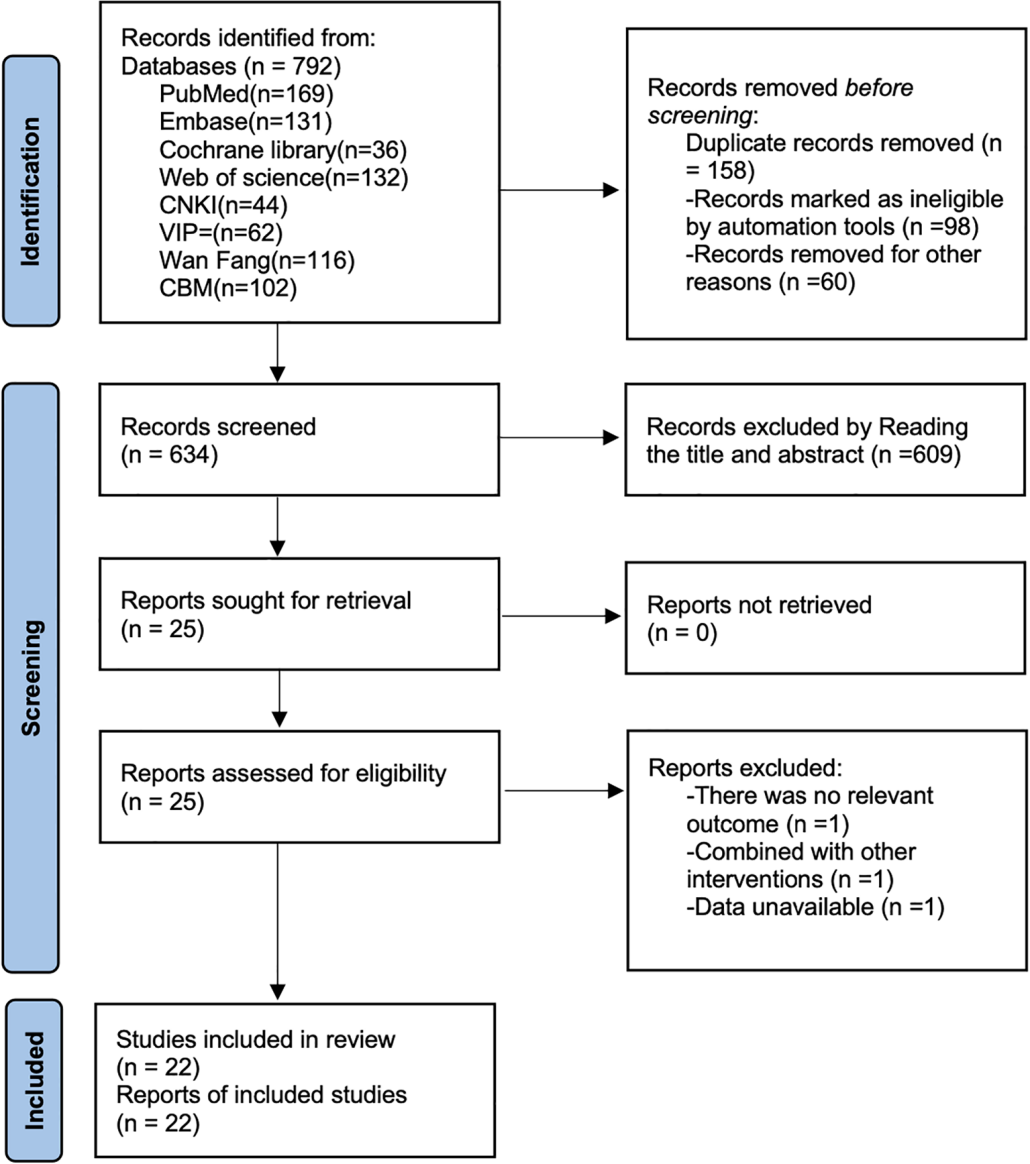
Literature search flowchart.

As shown in **Figure 1**, a total of 792 articles were retrieved by searching PubMed (n=169), Embase (n=131), Cochrane library (n=36), Web of science (n=132), CNKI (n=44), VIP (n=62), CBM (n=102) and Wan Fang (n=116) through the removing 158 duplicates, 609 articles by reading the title and abstract, and 3 articles by reading the full text, and finally 22 randomized controlled studies (34–55) were included.

### Basic characteristics of literature

A total of 22 randomized controlled studies involving 1827 patients including Qigong (n=1), Taichi (n=3), Baduanjin (n=11), Liuzijue (n=5) and Wuqinxi (n=2) with a mean age range of 55–68 were included. Specific basic characteristics are shown in **Table 1**.

**Table 1:** Basic characteristics of the included literature.

Study	Year	Sample size		Gender(M/F)	Disease stage	Treatment context	Cancer type	Adherence	Mean age(years)		Intervention		Outcome
		EG	CG						EG	CG	EG	CG	
Cheung (34)	2021	9	11	11/9	Advanced	Chemotherapy	NSCLC	100%	61.11	58.36	Tai chi 60min, 3/week, 12weeks	standard treatment	F1; F2; F3; F4;
Gu (35)	2025	45	45	48/42	Early	Post-surgery	Adenocarcinoma Squamous carcinoma small cell carcinoma	90%	61.17	59.82	Liuzijue: 30min; 2/week, 8weeks	standard treatment	F4; F5
Jiang (36)	2020	50	50	56/44	Early	Post-surgery	NSCLC	100%	59.3	57.56	Tai chi 60min, 3/week, 12weeks	standard treatment	F6; F7
Liu (37)	2024	36	37	50/23	Advanced	Chemotherapy	NSCLC	85%	57	56	Baduanjin:20min; 3day	standard treatment	F1; F2; F3
Molassiotis (38)	2021	78	78	116/40	Advanced	Chemotherapy	NSCLC	87%	57.62	56.06	Qigong: 90min; 2week	standard treatment	F1
Wu (39)	2025	32	32	38/25	Early	Chemotherapy	NR	99%	66.29	66.38	Baduanjin:20min; 3day	standard treatment	F3
Xu (40)	2023	102	103	NR	Advanced	Chemotherapy	NSCLC	100%	65.4	63.1	Baduanjin:20min; 3day	standard treatment	F4; F6; F7
Zhang (41)	2013	13	14	15/12	Early	Chemotherapy	NSCLC	96%	63.07	59.27	Tai chi 60min, 3/week, 12weeks	standard treatment	F8
JP Yu (42)	2024	50	50	75/25	Advanced	Chemotherapy	NSCLC	100%	67.73	67.17	Wuqinxi: 60min, 3/week, 12weeks	standard treatment	F1; F2; F4; F8
L Guan (43)	2019	30	30	35/25	Early	Post-surgery	NR	100%	63.41	63.42	Baduanjin:20min; 3/week, 6weeks	standard treatment	F1; F2; F3
SS Liu (44)	2021	39	39	49/29	Advanced	Chemotherapy	NR	100%	54.75	54.89	Baduanjin:20min; 3/week, 12weeks	standard treatment	F1; F2; F5
CH Ying (45)	2024	58	58	73/43	Advanced	Post-surgery	NSCLC	100%	63.36	61.36	Baduanjin:20min; 3/week, 6weeks	standard treatment	F1; F2; F4; F5
SJ Zhang (46)	2024	50	50	NR	Advanced	Chemotherapy	NSCLC	100%	NR	NR	Baduanjin:20min; 3/week, 12weeks	standard treatment	F4
QY Zhang (47)	2024	43	43	48/38	Advanced	Post-surgery	NR	100%	63.48	65.85	Baduanjin:20min; 3/week, 12weeks	standard treatment	F6; F7
Q Li (48)	2017	35	35	38/29	Advanced	Post-surgery	NSCLC	100%	56.26	56.09	Baduanjin:20min; 3day	standard treatment	F1; F2; F4
C Yang (49)	2021	30	30	31/29	Advanced	Post-surgery	NR	100%	55.26	57.17	Liuzijue: 30min; 2/week, 8weeks	standard treatment	F1; F2; F6; F7
J Wang (50)	2022	30	30	36/24	Advanced	Post-surgery	NR	100%	59.12	62.12	Liuzijue: 30min; 2/week, 8weeks	standard treatment	F6; F7
LY Wang (51)	2022	30	30	37/23	Advanced	Post-surgery	NSCLC	100%	58.35	59.55	Baduanjin:20min; 3/week, 12weeks	standard treatment	F6; F7
H Su (52)	2024	31	31	33/29	Advanced	Chemotherapy	NR	100%	59.65	59.87	Liuzijue: 30min; 2/week, 8weeks	standard treatment	F4; F6; F7
N Zhao (53)	2024	60	60	83/37	Advanced	Post-surgery	NR	100%	61.43	62.13	Baduanjin:30min; 3/week, 8weeks	standard treatment	F1; F2; F4; F6
YW Chen (54)	2019	30	30	44/16	Advanced	Chemotherapy	NR	100%	61.1	61.87	Wuqinxi: 60min, 3/week, 12weeks	standard treatment	F4; F5
R Han (55)	2016	30	30	28/32	Advanced	Chemotherapy	NSCLC	100%	54.9	57.1	Baduanjin:30min; 3/week, 8weeks	standard treatment	F4; F5; F6; F7

EG, Experimental group; CG, Control group; F1: anxiety; F2: depression; F3: PSQI, Pittsburgh Sleep Quality Index; F4: 6MWT, 6-minute-walk-test; F5: Quality of Life; F6: FEV1; F7: FVC; F8:CD4+/CD8+; NSCLC, non- small-cell lung cancer; NR, not reported.

### Risk of bias results

Twenty-two studies included in this study were evaluated for quality using risk of bias, of which 20 articles accounted for the specific method used for randomization process and were therefore evaluated as low risk. 16 articles referred to the deviations from intended interventions used and were therefore evaluated as low risk, and specific quality evaluations can be found in **Supplementary Material Figures S1**, **S2**.

### Meta- analysis results

#### Anxiety score

**Figure 2:**
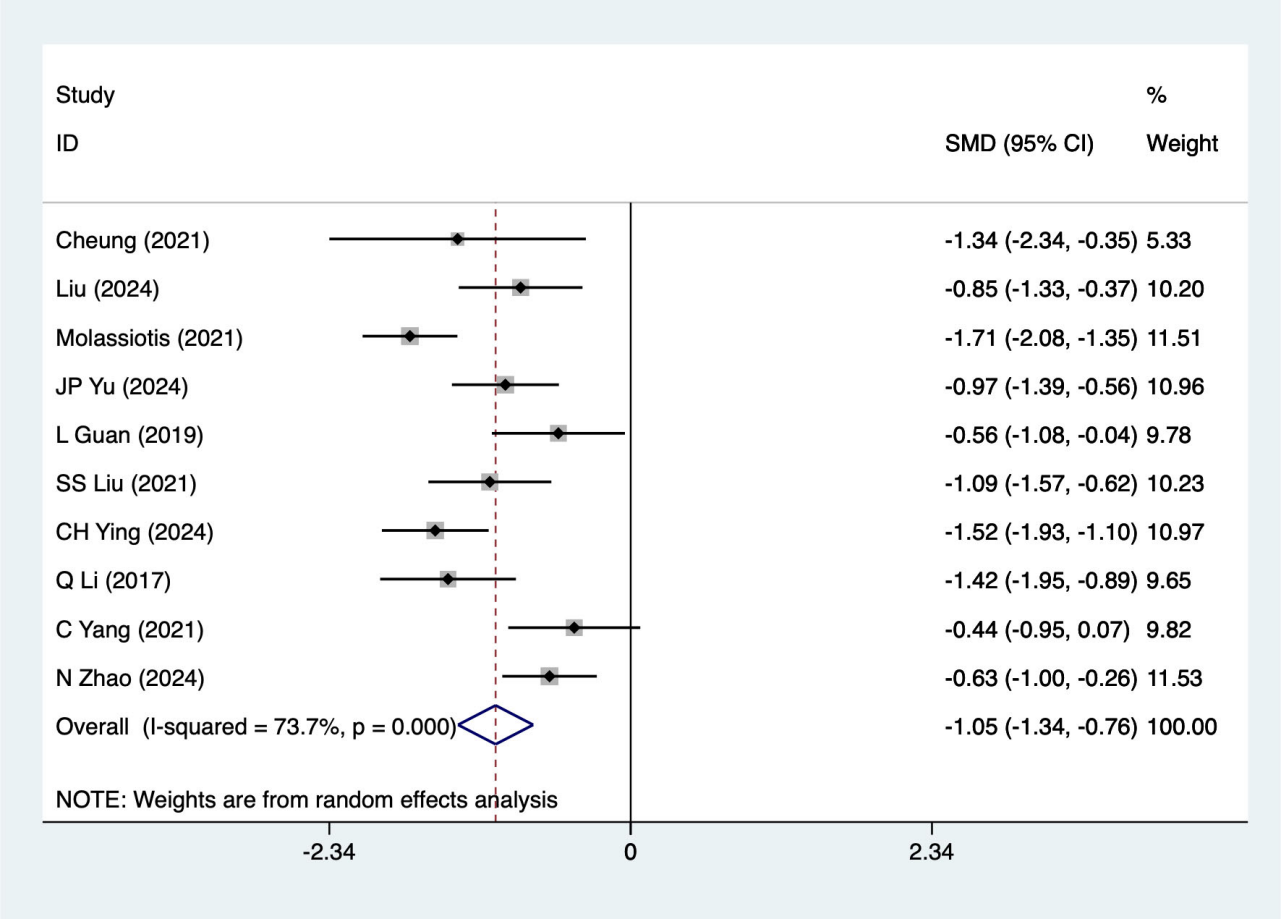
Meta-analysis of effects of mind-body traditional Chinese exercise on anxiety scores.

Anxiety scores were mentioned in 10 studies, tested for heterogeneity (*I^2^* = 73.7%, *P* = 0.001), and analyzed using a random-effects model, and the results of the analyses (**Figure 2**) suggested that mind-body traditional Chinese exercises were able to reduce anxiety scores in lung cancer patients [SMD=-1.05, 95%CI (- 1.34, -0.76)]. Due to the large heterogeneity, a sensitivity analysis was performed using a case-by-case exclusion, and the results of the analysis (**Supplementary Material Figure S3**) suggested that the results of this metric analysis were stable and unaffected by a single study. Subgroup analyses results (**Table 2**) suggested that Baduanjin [SMD=-1.01, 95%CI (-1.34, -0.68)], Advanced stage [SMD=-1.10,95%CI (-1.41, -0.80)], Chemotherapy [SMD=-1.19,95%CI (-1.56, -0.83)], Post-surgery [SMD=-0.92,95%CI (-1.36, -0.47)] mind-body traditional Chinese exercises reduced anxiety scores, other subgroup results should be considered exploratory and interpreted with caution due to the limited number of studies.

**Table 2:** Results of meta subgroup analysis.

Outcomes	Group	Subgroup	No of study	Heterogeneity		SMD (95%CI)
				I^2^(%)	P	
Anxiety score	Types of intervention	Taichi	1	NA	NA	-1.34(-2.43, -0.35)
		Baduanjin	6	67.9	0.008	-1.01(-1.34, -0.68)
		Qigong	1	NA	NA	-1.71(-2.08, -1.35)
		Wuqinxi	1	NA	NA	-0.97(-1.39, -0.56)
		Liuzijue	1	NA	NA	-0.44(-0.95, 0.07)
	Cancer stage	Advanced	9	73.5	0.001	-1.10(-1.41, -0.80)
		Early	1	NA	NA	-0.56(-1.08, -0.04)
	treatment context	Chemotherapy	5	63	0.029	-1.19(-1.56, -0.83)
		Post-surgery	5	79	0.001	-0.92(-1.36, -0.47)
Depression score	Types of intervention	Taichi	1	NA	NA	-1.22(-2.19, -0.24)
		Baduanjin	5	82	0.001	-1.29(-1.77, -0.80)
		Wuqinxi	1	NA	NA	-1.99(-2.45, -1.51)
		Liuzijue	1	NA	NA	-0.39(-0.90, 0.13)
	Cancer stage	Advanced	8	83.4	0.001	-1.26(-1.69, -0.82)
		Early	0	NA	NA	NA
	treatment context	Chemotherapy	4	65.6	0.033	-1.37(-1.85, -0.89)
		Post-surgery	4	90.4	0.001	-1.16(-1.90, -0.42)
Pittsburgh Sleep Quality Index	Types of intervention	Taichi	1	NA	NA	-0.25(-1.14, 0.63)
		Baduanjin	3	75.4	0.017	-0.76(-1.35, -0.17)
	Cancer stage	Advanced	2	71.3	0.062	-1.24(-2.82, 0.33)
		Early	2	54.8	0.137	-1.01(-1.86, -0.16)
	treatment context	Chemotherapy	3	47.9	0.147	-0.48(-0.94, -0.01)
		Post-surgery	1	NA	NA	-1.25(-1.81, -0.70)
6-minute-walk-test (WMD)	Types of intervention	Taichi	1	NA	NA	30.02(-0.78, 60.82)
		Liuzijue	2	0	0.690	47.45(9.53, 85.37)
		Baduanjin	6	45.9	0.100	31.36(19.60, 43.12)
		Wuqinxi	2	94	0.001	25.46(-10.38, 61.30)
	Cancer stage	Advanced	10	72.7	0.001	30.12(18.11, 42.13)
		Early	1	NA	NA	50.62(9.62, 91.62)
	treatment context	Chemotherapy	7	68.9	0.004	25.35(10.28, 40.42)
		Post-surgery	4	58.9	0.063	39.68(22.83, 56.53)
Quality of Life	Types of intervention	Liuzijue	1	NA	NA	0.38(-0.04, 0.79)
		Baduanjin	3	76.7	0.014	0.75(0.20, 1.29)
		Wuqinxi	1	NA	NA	1.73(1.13, 2.33)
	Cancer stage	Advanced	4	83.4	0.001	0.98(0.38, 1.57)
		Early	1	NA	NA	0.38(-0.04, 0.79)
	treatment context	Chemotherapy	3	81.9	0.004	1.16(0.44, 1.89)
		Post-surgery	2	0	0.724	0.43(0.16, 0.71)
FEV1 (WMD)	Types of intervention	Taichi	1	NA	NA	0.21(0.13, 0.29)
		Baduanjin	3	0	0.373	0.05(0.03, 0.08)
		Liuzijue	4	59.3	0.061	0.16(0.01, 0.30)
	Cancer stage	Advanced	7	64	0.011	0.12(0.03, 0.21)
		Early	1	NA	NA	0.21(0.13, 0.29)
	treatment context	Chemotherapy	3	0	0.952	0.05(0.03, 0.07)
		Post-surgery	5	9.9	0.350	0.20(0.14, 0.27)
FVC (WMD)	Types of intervention	Taichi	1	NA	NA	0.31(0.14, 0.48)
		Baduanjin	3	71.1	0.031	0.10(-0.10, 0.31)
		Liuzijue	4	0	0.483	0.07(-0.02, 0.16)
	Cancer stage	Advanced	7	68.2	0.004	0.05(0.00, 0.10)
		Early	1	NA	NA	0.31(0.14, 0.48)
	treatment context	Chemotherapy	3	0	0.772	-0.01(-0.03, 0.02)
		Post-surgery	5	80.9	0.001	0.18(0.04, 0.31)

#### Depression score

**Figure 3:**
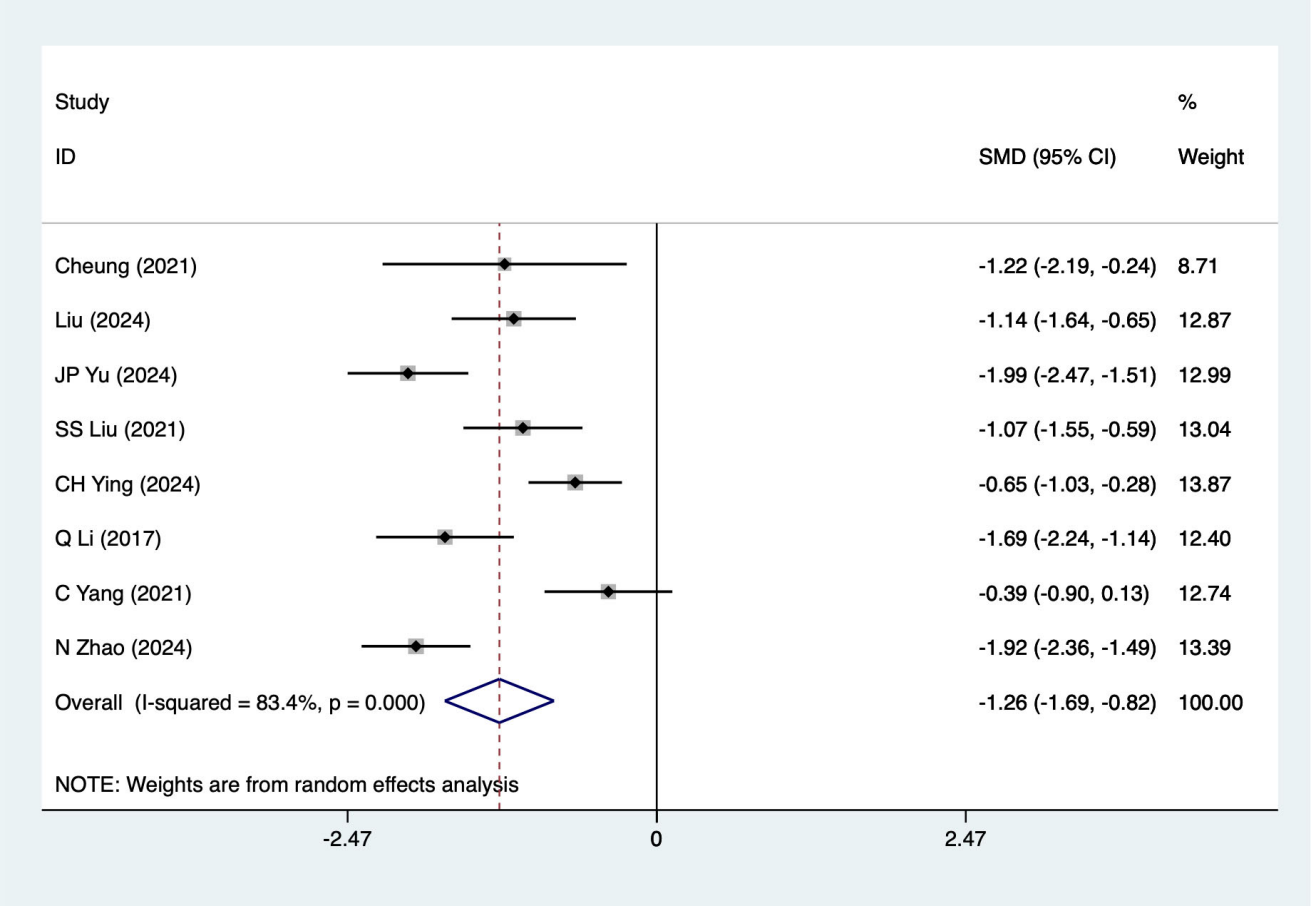
Meta-analysis of effects of mind-body traditional Chinese exercise on depression scores.

Depression score were mentioned in 8 studies, tested for heterogeneity *(I^2^* = 83.4%, *P* = 0.001), and analyzed using a random-effects model, and the results of the analyses (**Figure 3**) suggested that mind-body traditional Chinese exercises were able to reduce depression scores in lung cancer patients [SMD=-1.05, 95%CI (- 1.26, -0.82)]. Due to the large heterogeneity, a sensitivity analysis was performed using a case-by-case exclusion, and the results of the analysis (**Supplementary Material Figure S4**) suggested that the results of this metric analysis were stable and unaffected by a single study. Subgroup analyses results suggested that Baduanjin [SMD=-1.29, 95%CI (-1.77, -0.80)], Advanced stage [SMD=-1.26, 95%CI (-1.69, -0.82)], Chemotherapy [SMD=-1.37, 95%CI (-1.85, -0.89)], Post-surgery [SMD=-1.16, 95%CI (-1.90, -0.42)], mind-body traditional Chinese exercises reduced depression scores, other subgroup results should be considered exploratory and interpreted with caution due to the limited number of studies.

#### PSQI

**Figure 4:**
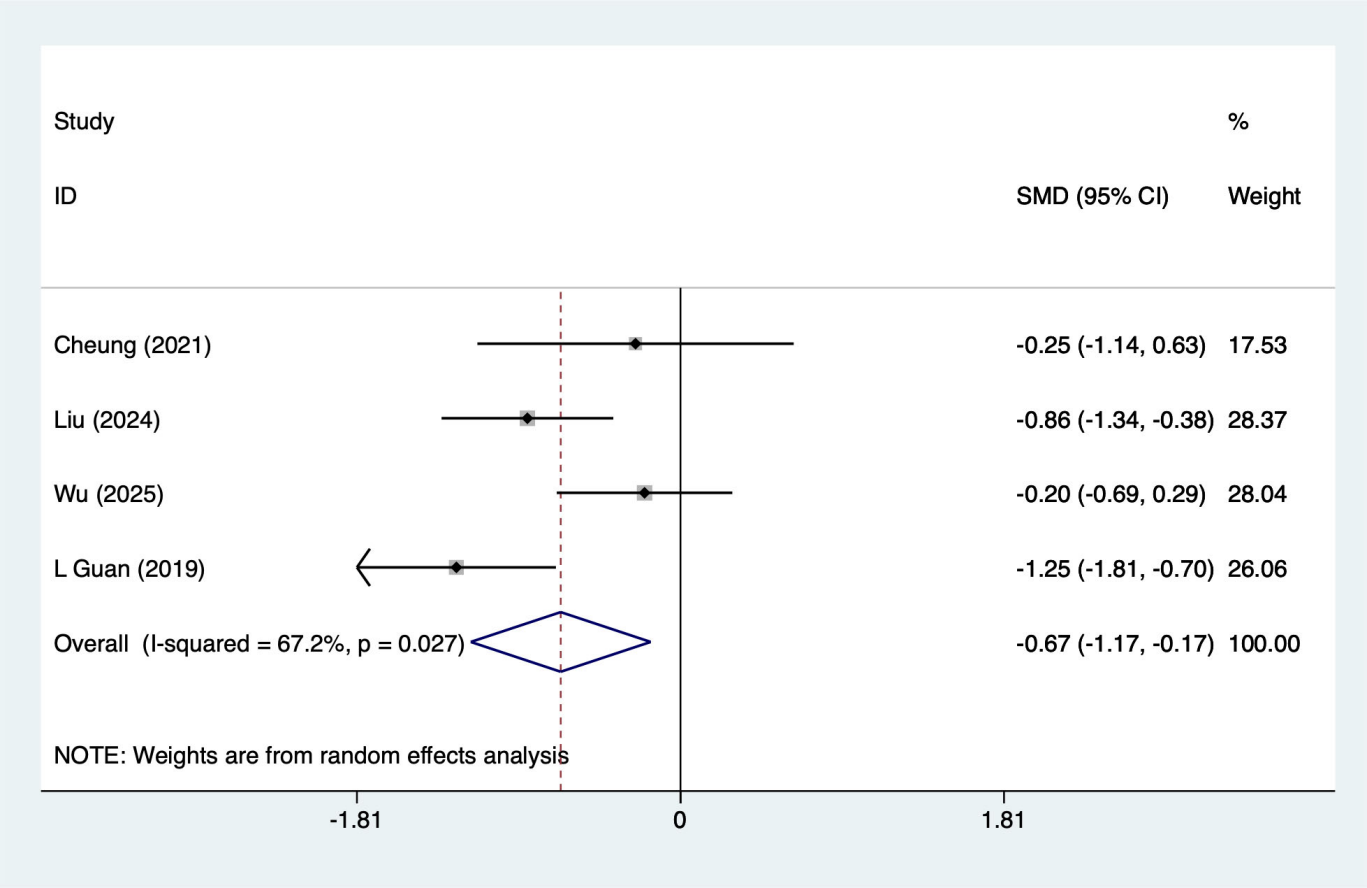
Meta-analysis of effects of mind-body traditional Chinese exercise on PSQI scores.

PSQI were mentioned in 4 studies, tested for heterogeneity (*I^2^* = 67.2%, *P* = 0.027), and analyzed using a random-effects model, and the results of the analyses (**Figure 4**) suggested that mind-body traditional Chinese exercises were able to reduce PSQI scores in lung cancer patients [SMD=-0.67, 95%CI (-1.17, -0.17)]. Due to the large heterogeneity, a sensitivity analysis was performed using a case-by-case exclusion, and the results of the analysis (**Supplementary Material Figure S5**) suggested that the results of this metric analysis were stable and unaffected by a single study. Subgroup analyses results suggested that Baduanjin [SMD=-0.76, 95%CI (-1.35, -0.17)] may reduce PSQI scores, other subgroup results should be considered exploratory and interpreted with caution due to the limited number of studies.

#### 6MWT

**Figure 5:**
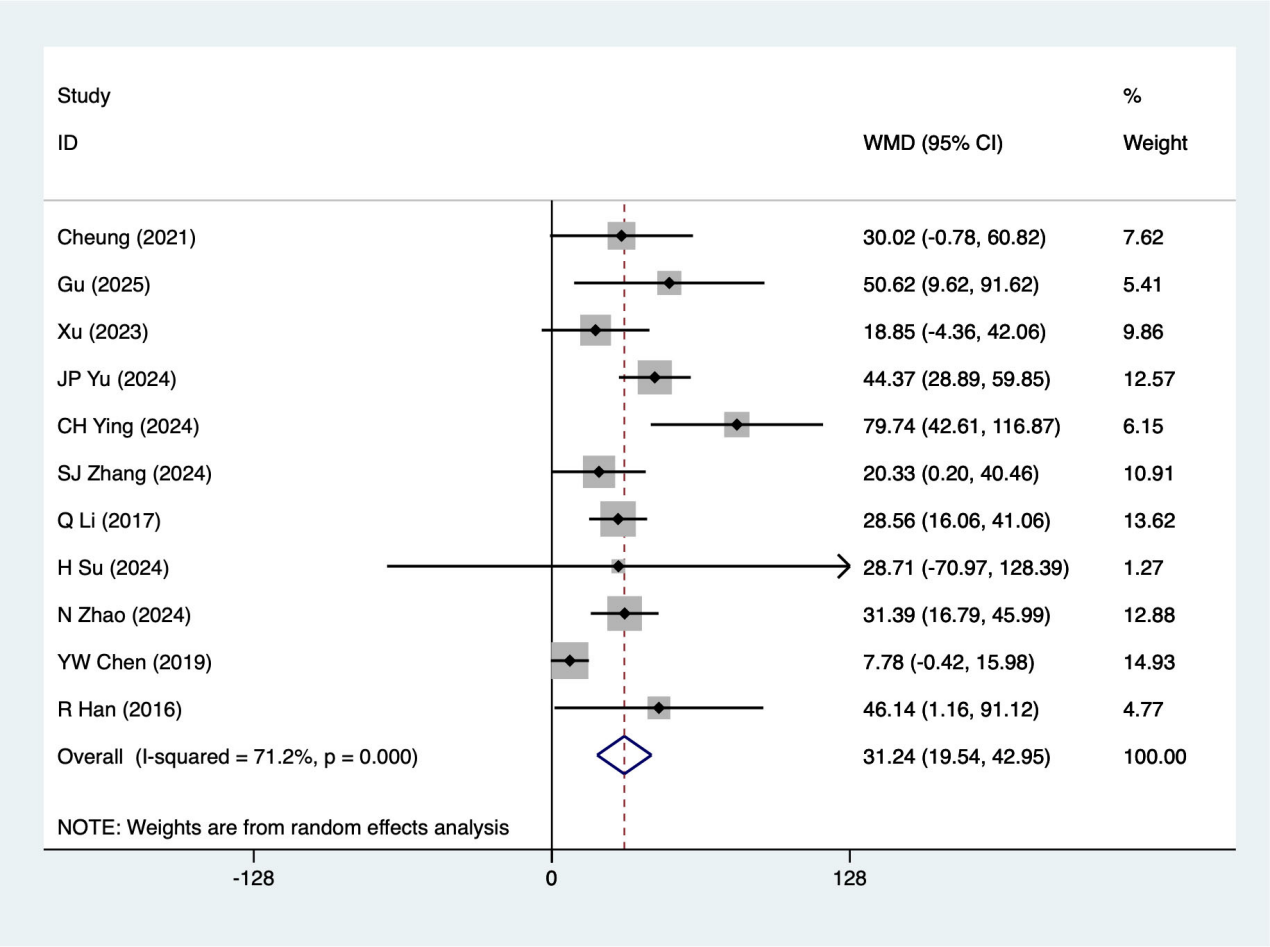
Meta-analysis of effects of mind-body traditional Chinese exercise on 6MWT.

6MWT were mentioned in 11 studies, tested for heterogeneity (*I^2^* = 71.2%, *P* = 0.001), and analyzed using a random-effects model, and the results of the analyses (**Figure 5**) suggested that mind-body traditional Chinese exercises were able to increase 6MWT in lung cancer patients [WMD = 31.24, 95%CI (19.54, 42.95)]. Due to the large heterogeneity, a sensitivity analysis was performed using a case-by-case exclusion, and the results of the analysis (**Supplementary Material Figure S6**) suggested that the results of this metric analysis were stable and unaffected by a single study. The subgroup analysis showed statistically significant differences in the Baduanjin [WMD = 31.36, 95%CI (19.60, 43.12)], Advanced stage [WMD = 30.12, 95%CI (18.11, 42.13)], Chemotherapy [WMD = 25.35, 95%CI (10.28, 40.42)], Post-surgery [WMD = 39.68, 95%CI (22.83, 56.53)], other subgroup results should be considered exploratory and interpreted with caution due to the limited number of studies. Although these subgroups are statistically significant, their clinical significance is limited. Readers should therefore interpret these results with caution.

#### Quality of Life

**Figure 6:**
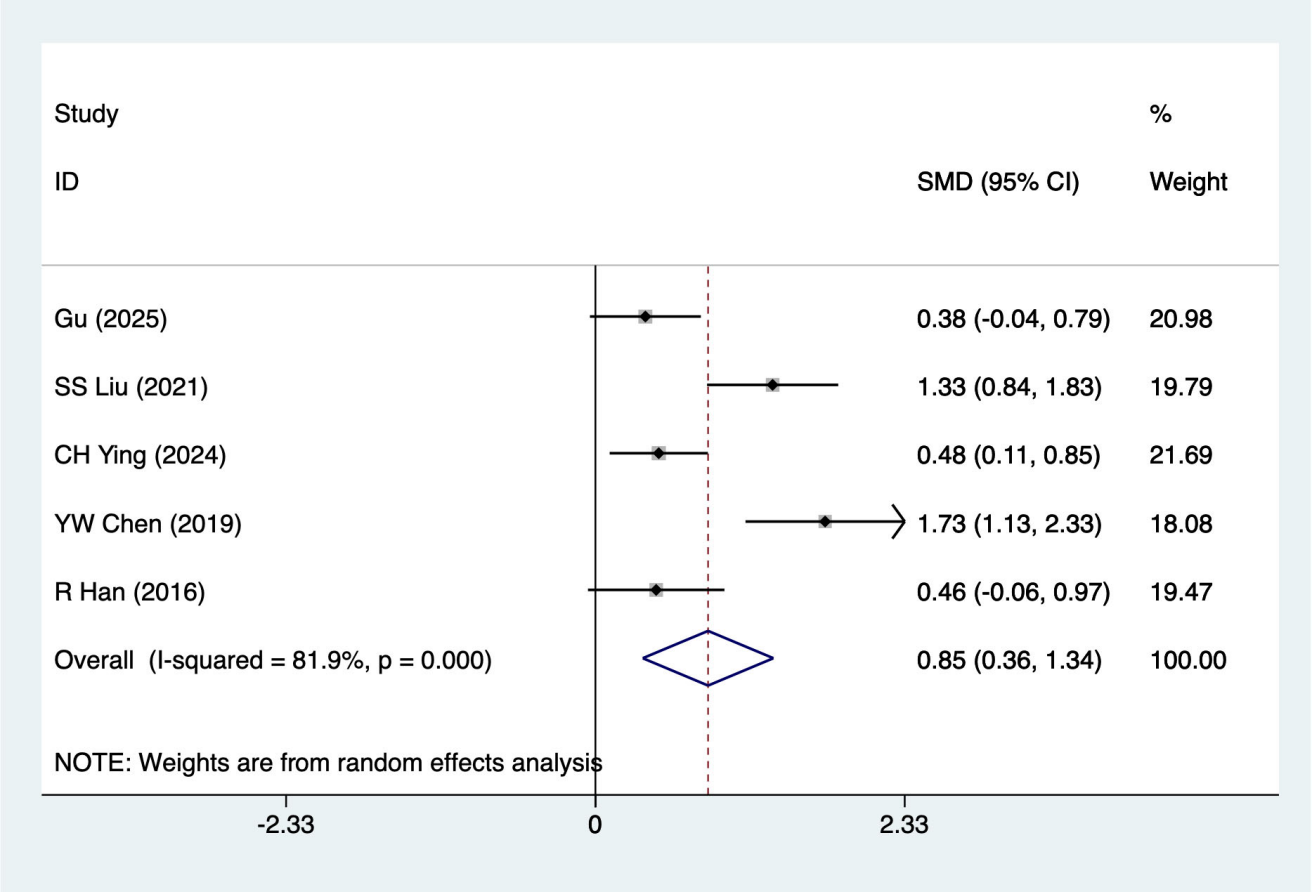
Meta-analysis of effects of mind-body traditional Chinese exercise on quality of life.

Quality of Life were mentioned in 5 studies, tested for heterogeneity (*I^2^* = 81.9%, *P* = 0.001), and analyzed using a random-effects model, and the results of the analyses (**Figure 6**) suggested that mind-body traditional Chinese exercises were able to increase quality of life scores in lung cancer patients [SMD = 0.85, 95%CI (0.36, 1.34)]. Due to the large heterogeneity, a sensitivity analysis was performed using a case-by-case exclusion, and the results of the analysis (**Supplementary Material Figure S7**) suggested that the results of this metric analysis were stable and unaffected by a single study. Subgroup analyses results suggested that Baduanjin [SMD = 0.75, 95%CI (0.20, 1.29)], Advanced stage [SMD = 0.98, 95%CI (0.38, 1.57)], Chemotherapy [SMD = 1.16, 95%CI (0.44, 1.89)], mind-body traditional Chinese exercises increase quality of life scores, other subgroup results should be considered exploratory and interpreted with caution due to the limited number of studies.

#### FEV1

**Figure 7:**
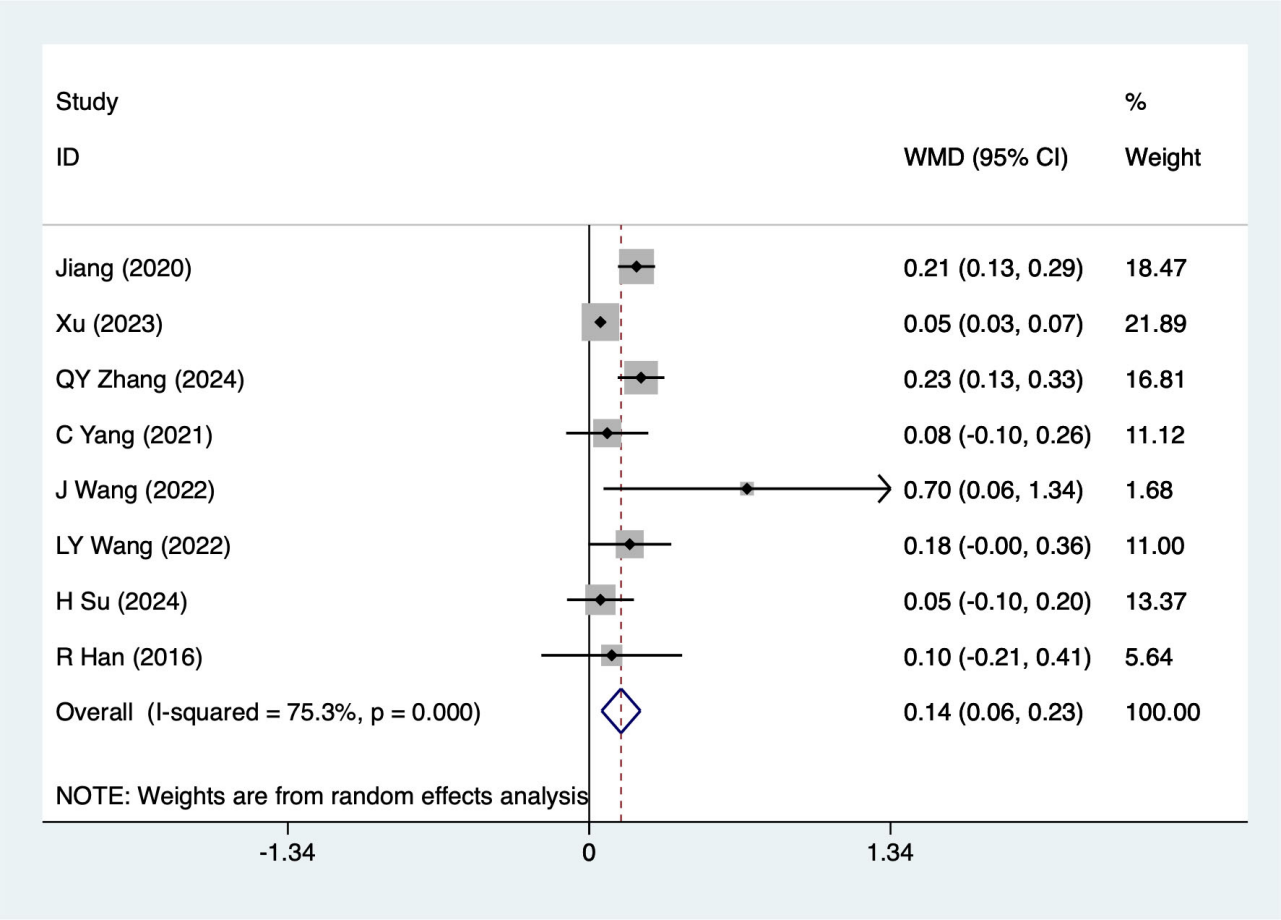
Meta-analysis of effects of mind-body traditional Chinese exercise on FEV1.

FEV1 were mentioned in 8studies, tested for heterogeneity (*I^2^* = 75.3%, *P* = 0.001), and analyzed using a fixed-effects model, and the results of the analyses (**Figure 7**) suggested that mind-body traditional Chinese exercises were able to increase FEV1 in lung cancer patients [WMD = 0.14, 95%CI (0.06, 0.23)]. The subgroup analysis showed statistically significant differences in the Baduanjin [WMD = 0.05, 95%CI (0.03, 0.08)], Liuzijue[WMD = 0.16, 95%CI (0.01, 0.30)], for advanced stage lung cancer [WMD = 0.12, 95%CI (0.03, 0.21)], Combination chemotherapy [WMD = 0.05, 95%CI (0.03, 0.07)], for post-surgery lung cancer [WMD = 0.20, 95%CI (0.14, 0.27)], other subgroup results should be considered exploratory and interpreted with caution due to the limited number of studies. Although these subgroups are statistically significant, their clinical significance is limited. Readers should therefore interpret these results with caution.

#### FVC

**Figure 8:**
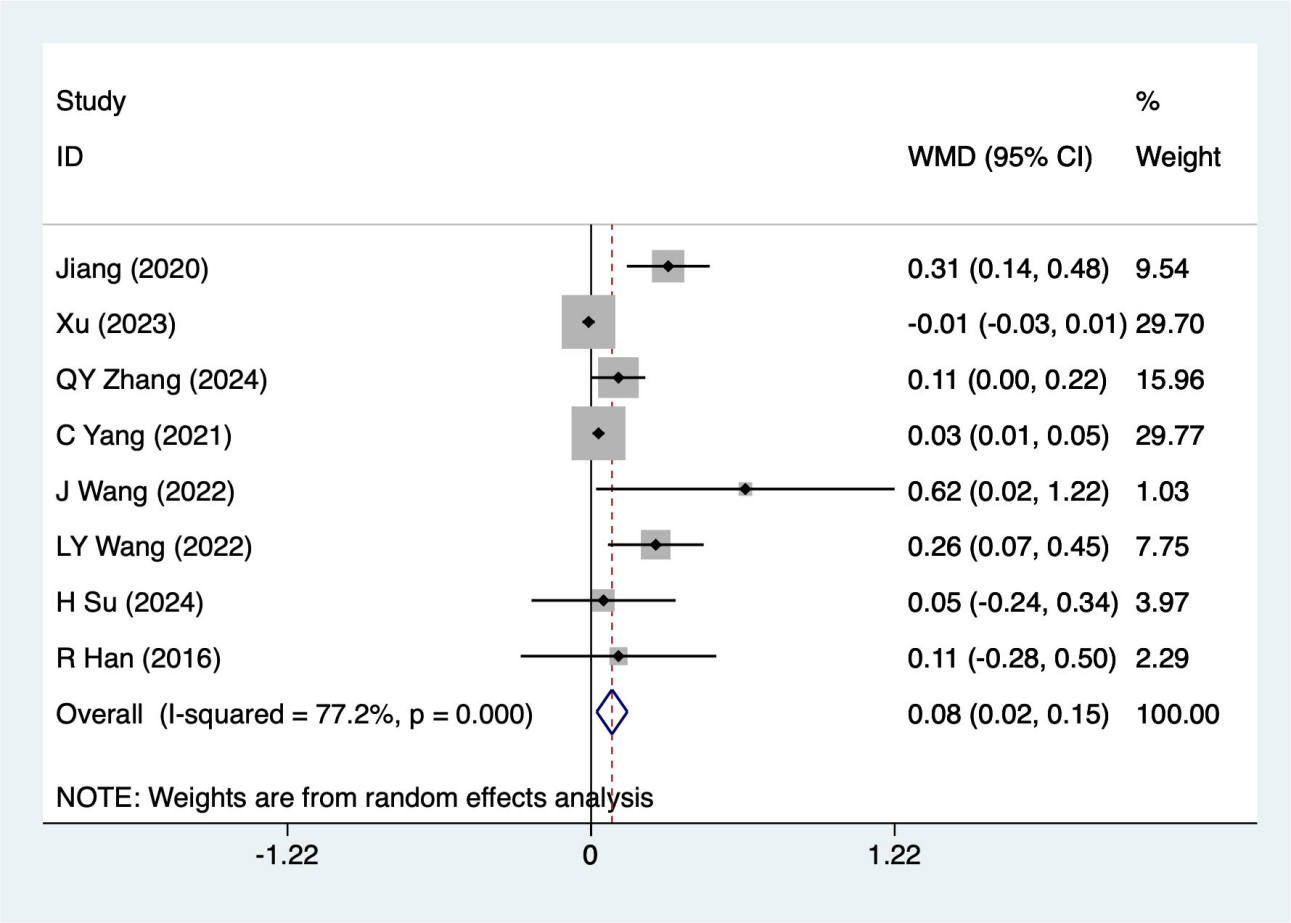
Meta-analysis of effects of mind-body traditional Chinese exercise on FVC.

FVC were mentioned in 8studies, tested for heterogeneity (*I^2^* = 77.2%, *P* = 0.001), and analyzed using a random-effects model, and the results of the analyses (**Figure 8**) suggested that mind-body traditional Chinese exercises were able to increase FVC in lung cancer patients [WMD = 0.08, 95%CI (0.02, 0.15)]. Due to the large heterogeneity, a sensitivity analysis was performed using a case-by-case exclusion, and the results of the analysis (**Supplementary Material Figure S8**) suggested that the results of this metric analysis were stable and unaffected by a single study. Subgroup analyses results suggested that for Post-surgery [WMD = 0.18, 95%CI (0.04, 0.31)], mind-body traditional Chinese exercises increase FEV1, other subgroup results should be considered exploratory and interpreted with caution due to the limited number of studies.

### Publication bias

In this study, the egger test and funnel plot were used for the detection of publication bias, and the results (**Supplementary Material Figures S8**-**S14**) suggested that the funnel plot was symmetrical, and the results of the egger test were all P > 0.05, suggesting that there was a low likelihood of publication bias.

### Meta- regression

Because of the large heterogeneity in outcomes, meta-regression was used to explore sources of heterogeneity, and the results (**Supplementary Material Figures S15**-**21**; **Table 3**) suggest that tumor type and intervention were not sources of heterogeneity.

**Table 3:** Table of meta-regression results.

Outcomes	Group	P
Anxiety score	Intervention	0.87
	Cancer type	0.13
Depression score	Intervention	0.07
	Cancer type	0.09
Pittsburgh Sleep Quality Index	Intervention	0.13
	Cancer type	0.83
Quality of Lif	Intervention	0.19
	Cancer type	0.78
FEV1	Intervention	0.49
	Cancer type	0.53
FVC	Intervention	0.91
	Cancer type	0.34

## Discussion

The results of this study showed that TCM mind-body exercises (Qigong, Taichi, Baduanjin, Liuzijue and Wuqinxi) showed significant efficacy in improving anxiety, depression, quality of sleep, exercise capacity and quality of life in lung cancer patients. Compared to similar meta-analyses (56), the diversity of intervention methods in this study—encompassing multiple traditional Chinese medicine fitness practices such as qigong and tai chi—not only improved lung function and exercise capacity in lung cancer patients but also significantly enhanced multiple aspects including anxiety, depression, sleep quality, and quality of life. Moreover, its multidimensional assessment approach enhances the intervention’s comprehensive and practical utility in clinical settings. Notably, traditional Chinese fitness methods demonstrated markedly positive effects on mental health.

Meta-analysis of this study showed that TCM physical and mental exercises significantly reduced anxiety and depression symptoms in lung cancer patients. Subgroup analyses revealed that different interventions such as Baduanjin was effective in reducing patients’ anxiety and depression scores, anxiety and depression often accompany cancer patients, especially lung cancer patients, and these mood disorders may be exacerbated by cancer-related symptoms, treatment side effects, and disease progression (27). TCM mind-body exercises such as Tai Chi and Qigong regulate the autonomic nervous system through a combination of deep breathing, meditation, and slow movements, and can effectively activate the parasympathetic nervous system, thereby reducing the stress response in the body (57). Specifically, mind-body exercises can help reduce anxiety and depressive symptoms by modulating neurotransmitters in the brain, such as increasing the secretion of endorphins and serotonin. Additionally, the meditative component of qigong and tai chi may further enhance emotional stability and mental resilience by improving self-awareness and emotional regulation.

This study also demonstrated that TCM mind-body exercises can significantly improve sleep quality in lung cancer patients, especially Baduanjin. Improvement in sleep quality is not only a sign of physical recovery, but also an important indicator of psychological health. Lung cancer patients often face sleep disorders due to pain, treatment side effects, or psychological stress (58). Physical and mental exercises such as Baduanjin can promote sleep by regulating the autonomic nervous system, especially through deep breathing exercises, which can relax the patient’s body and reduce the excitation of the sympathetic nervous system (34). Deep abdominal breathing increases oxygen intake and improves blood oxygen levels, further promoting sleep quality (59). These exercises also indirectly help patients sleep better by reducing anxiety and improving emotional regulation.

Our study showed that TCM mind–body exercises significantly increased the exercise capacity of lung cancer patients, particularly as reflected in improvements in the 6MWT. Overall, these traditional exercises may enhance exercise endurance by strengthening musculature, improving pulmonary ventilation, and optimizing cardiopulmonary function (60). TCM mind–body exercises and other coordinated movement–breathing exercises can increase cardiac output and improve the efficiency of pulmonary oxygen exchange through integrated movement control and abdominal breathing, thereby supporting better exercise performance. In addition, mind–body exercises may further enhance exercise capacity by increasing the strength of the pectoral muscles and diaphragm, promoting greater lung expansion, and reducing discomfort during physical activity (19).

Physical and mental exercises indirectly improve the patient’s quality of life by regulating the patient’s psychological state (reducing anxiety, depression and other emotions), improving the quality of sleep and enhancing physical strength (61). Exercises such as Baduanjin and Wuqinxi help patients restore normal daily function by improving their physical mobility. In addition, mind-body exercises enhance patients’ self-regulation through meditation and deep-breathing techniques, which help to alleviate the psychological burden brought about by cancer treatment and thus improve overall quality of life (62).

This study also found that TCM physical and mental exercises significantly improved lung function in lung cancer patients, particularly in the pooled FEV1 and FVC indices. Although some individual exercise modalities showed signals of potential benefit, these subgroup findings did not reach statistical significance and should be interpreted with caution. Overall, the improvement in lung function appears closely related to the deep-breathing components and targeted muscle engagement inherent in mind–body exercises (25). By increasing breathing depth and regulating respiratory rhythm, these practices may enhance lung ventilation and oxygen exchange and support better airway patency in lung cancer patients (63). Additionally, mind–body exercises can promote greater lung expandability and improve ventilation efficiency by strengthening respiratory muscles, thereby contributing to improvements in lung function indicators such as FEV1 and FVC (64).

In the subgroup analysis of this study, Baduanjin demonstrated overall favorable efficacy, showing significant positive effects in both early-stage and advanced-stage lung cancer patients. This suggests that Baduanjin could be a potentially effective intervention across different stages of the disease. However, for certain subgroups where only one study was available, even statistically significant results should be interpreted with caution. The limited number of studies in these subgroups means that the findings are based on a small sample size, which increases the risk of bias and variability in the results. Therefore, while these results are promising, further research with larger sample sizes and more robust study designs is necessary to confirm the efficacy of Baduanjin in these specific contexts.

This study found that factors such as disease stage and concurrent treatments may have influenced the observed outcomes to varying degrees. First, anxiety and depression symptoms improved more significantly in advanced lung cancer patients after traditional Chinese posture exercises, likely due to their higher psychological burden, suggesting greater sensitivity to the intervention. Second, chemotherapy patients demonstrated particularly pronounced improvements in mental health and quality of life, likely due to the physiological and psychological stress induced by chemotherapy, which the traditional Chinese posture exercises helped alleviate. Additionally, postoperative patients exhibited marked improvements in lung function, indicating the intervention’s contribution to postoperative recovery. Therefore, future research should further explore how the frequency, duration, and specific form of the intervention affect its efficacy.

### Clinical significance

The results of this study provide strong evidence for the clinical application of TCM physical and mental exercises in lung cancer patients. Considering that lung cancer patients often face multiple physiological and psychological disturbances during the treatment process, TCM physical and mental exercises, as an adjuvant treatment, can effectively improve patients’ psychological state, exercise ability, sleep quality and quality of life, and have high clinical application value. It is recommended that clinicians develop a personalized treatment plan based on the patient’s individual situation, combining exercises such as Qigong, Taichi, Baduanjin, and Wuqinxi. These exercises not only provide patients with the opportunity to relax physically and mentally but also help to increase treatment compliance and improve quality of life.

### Strengths and limitations

This study assessed the effects of TCM mind-body exercises on multifaceted health indicators in lung cancer patients through a systematic Meta-analysis, providing high-quality evidence to support its application in the comprehensive treatment of lung cancer.

First, the sample size for some included studies was small, intervention frequency and duration varied, and most lung cancer pathologies included were non-small cell lung cancer. These factors may compromise the robustness and generalizability of the findings. In addition, important clinical variables such as disease stage (early vs. advanced), treatment context (pre-surgery, post-surgery, chemotherapy, radiotherapy, immunotherapy), and adherence to interventions (% of sessions attended) were not consistently reported or analyzed, which may further limit the interpretability and generalizability of the findings. Second, despite the use of randomized controlled trials, there was a high degree of heterogeneity between studies, especially in mental health indicators such as anxiety and depression, which may be affected by different treatment modalities and individual patient differences. Moreover, there was a lack of standardization in some of the interventions, which may have affected the accuracy and consistency of the results. Finally, the included studies lacked long-term follow-up data, limiting the ability to comprehensively assess the impact of physical and mental exercises on the long-term prognosis and quality of survival of lung cancer patients.

## Conclusion

In this study, the results showed that the mind-body traditional Chinese exercises maybe significantly improve the patients’ anxiety, depression, sleep quality, exercise capacity, lung function and quality of life. These exercises have good prospects for clinical application and can effectively improve the overall health of patients as an adjunctive treatment in the comprehensive treatment of lung cancer. However, due to the heterogeneity of the studies and the limitation of the sample size, their long-term efficacy and safety need to be further verified by larger, standardized randomized controlled trials in the future.

## Data Availability

The original contributions presented in the study are included in the article/**Supplementary Material**. Further inquiries can be directed to the corresponding author.

## References

[1] Kalemkerian GP Khurshid H Ismaila N . Systemic therapy for small cell lung cancer: ASCO guideline rapid recommendation update. J Clin Oncol. (2025) 43:101–5. doi: 10.1200/JCO-24-02245, PMID: 39565968

[2] Sung H Ferlay J Siegel RL Laversanne M Soerjomataram I Jemal A . Global cancer statistics 2020: GLOBOCAN estimates of incidence and mortality worldwide for 36 cancers in 185 countries. CA Cancer J Clin. (2021) 71:209–49. doi: 10.3322/caac.21660, PMID: 33538338

[3] Qi J Li M Wang L Hu Y Liu W Long Z . National and subnational trends in cancer burden in China, 2005-20: an analysis of national mortality surveillance data. Lancet Public Health. (2023) 8:e943–e55. doi: 10.1016/S2468-2667(23)00211-6, PMID: 38000889

[4] Daum S Decristoforo L Mousa M Salcher S Plattner C Hosseinkhani B . Unveiling the immunomodulatory dance: endothelial cells’ function and their role in non-small cell lung cancer. Mol Cancer. (2025) 24:21. doi: 10.1186/s12943-024-02221-6, PMID: 39819502 PMC11737145

[5] Dong S Li X Huang Q Li Y Li J Zhu X . Resistance to immunotherapy in non-small cell lung cancer: Unraveling causes, developing effective strategies, and exploring potential breakthroughs. Drug Resist Updat. (2025) 81:101215. doi: 10.1016/j.drup.2025.101215, PMID: 40081220

[6] Simons E Camidge DR . Lung cancer oncogene-directed therapy, fertility, and pregnancy. J Thorac Oncol. (2024) 19:866–76. doi: 10.1016/j.jtho.2024.01.003, PMID: 38185202

[7] Meyer ML Fitzgerald BG Paz-Ares L Cappuzzo F Jänne PA Peters S . New promises and challenges in the treatment of advanced non-small-cell lung cancer. Lancet. (2024) 404:803–22. doi: 10.1016/S0140-6736(24)01029-8, PMID: 39121882

[8] Huang Q Li Y Huang Y Wu J Bao W Xue C . Advances in molecular pathology and therapy of non-small cell lung cancer. Signal Transduct Target Ther. (2025) 10:186. doi: 10.1038/s41392-025-02243-6, PMID: 40517166 PMC12167388

[9] Wang C Chen B Liang S Shao J Li J Yang L . China Protocol for early screening, precise diagnosis, and individualized treatment of lung cancer. Signal Transduct Target Ther. (2025) 10:175. doi: 10.1038/s41392-025-02256-1, PMID: 40425545 PMC12117065

[10] Jiao M Liang H Zhang M . Effect of exercise on postoperative recovery of patients with non-small cell lung cancer: a systematic review and meta-analysis. Discov Oncol. (2024) 15:230. doi: 10.1007/s12672-024-01079-w, PMID: 38884823 PMC11183035

[11] Hu Y Gu S Bu Z Liu Z Dong J Shi J . Effect of exercise for patients with advanced lung cancer and cancer-related fatigue: A systematic review and meta-analysis. J Sport Health Sci. (2024) 14:101017. doi: 10.1016/j.jshs.2024.101017, PMID: 39643115 PMC11910083

[12] Luo Z Wan R Liu S Feng X Peng Z Wang Q . Mechanisms of exercise in the treatment of lung cancer - a mini-review. Front Immunol. (2023) 14:1244764. doi: 10.3389/fimmu.2023.1244764, PMID: 37691942 PMC10483406

[13] Valenzuela PL Saco-Ledo G Santos-Lozano A Morales JS Castillo-García A Simpson RJ . Exercise training and natural killer cells in cancer survivors: current evidence and research gaps based on a systematic review and meta-analysis. Sports Med Open. (2022) 8:36. doi: 10.1186/s40798-022-00419-w, PMID: 35244811 PMC8897541

[14] Sun C Gao M Qiao M . Research progress of traditional Chinese medicine compound “Xiaochaihu Decoction” in the treatment of depression. BioMed Pharmacother. (2023) 159:114249. doi: 10.1016/j.biopha.2023.114249, PMID: 36682244

[15] Yan X Li F Dozmorov I Frank MB Dao M Centola M . External Qi of Yan Xin Qigong induces cell death and gene expression alterations promoting apoptosis and inhibiting proliferation, migration and glucose metabolism in small-cell lung cancer cells. Mol Cell Biochem. (2012) 363:245–55. doi: 10.1007/s11010-011-1176-8, PMID: 22160803 PMC3567610

[16] Tsai CH Chiang PH . More benefits of tai chi than aerobic exercise in patients with advanced lung cancer. JAMA Oncol. (2024) 10:1290–1. doi: 10.1001/jamaoncol.2024.2453, PMID: 38959005

[17] Faisal MAA Chowdhury MEH Khandakar A Hossain MS Alhatou M Mahmud S . An investigation to study the effects of Tai Chi on human gait dynamics using classical machine learning. Comput Biol Med. (2022) 142:105184. doi: 10.1016/j.compbiomed.2021.105184, PMID: 35016098

[18] Lei J Yang J Dong L Xu J Chen J Hou X . An exercise prescription for patients with lung cancer improves the quality of life, depression, and anxiety. Front Public Health. (2022) 10:1050471. doi: 10.3389/fpubh.2022.1050471, PMID: 36466452 PMC9714027

[19] Wu D Li J Dong H Zheng Y Cui H . The effect of Baduanjin on postoperative activity tolerance, lung function and negative emotions in patients with lung cancer: a systematic review and meta-analysis. Support Care Cancer. (2025) 33:631. doi: 10.1007/s00520-025-09690-5, PMID: 40571840

[20] Yin Z Martinez CE Li S Martinez M Peng K Land WM . Adapting Chinese Qigong mind-body exercise for healthy aging in older community-dwelling low-income Latino adults: pilot feasibility study. JMIR Aging. (2021) 4:e29188. doi: 10.2196/29188, PMID: 34723824 PMC8593812

[21] Qi A He Y Gu Y Zhang C Qin X Wang Y . Chinese herbal medicine combined with Liuzijue exercise in physiological rehabilitation after video-assisted lung lobectomy for cancer: A prospective propensity score matching study. Integr Cancer Ther. (2024) 23:15347354241261977. doi: 10.1177/15347354241261977, PMID: 38907709 PMC11193924

[22] Xiao CM Zhuang YC . Efficacy of Liuzijue Qigong in individuals with chronic obstructive pulmonary disease in remission. J Am Geriatr Soc. (2015) 63:1420–5. doi: 10.1111/jgs.13478, PMID: 26131612

[23] Su XE Hong WP He HF Xie B Wu SH . Impact of preoperative respiratory training on early postoperative recovery in patients undergoing lung cancer surgery: a randomized controlled trial. Support Care Cancer. (2025) 33:593. doi: 10.1007/s00520-025-09658-5, PMID: 40528050

[24] Takemura N Cheung DST Fong DYT Lee AWM Lam TC Ho JC . Effectiveness of aerobic exercise and tai chi interventions on sleep quality in patients with advanced lung cancer: A randomized clinical trial. JAMA Oncol. (2024) 10:176–84. doi: 10.1001/jamaoncol.2023.5248, PMID: 38060250 PMC10704344

[25] Hou W Zhai L Yang Y Gu S Li C Yang Y . Is physical activity effective against cancer-related fatigue in lung cancer patients? An umbrella review of systematic reviews and meta-analyses. Support Care Cancer. (2023) 31:161. doi: 10.1007/s00520-023-07627-4, PMID: 36781549

[26] Page MJ McKenzie JE Bossuyt PM Boutron I Hoffmann TC Mulrow CD . The PRISMA 2020 statement: an updated guideline for reporting systematic reviews. BMJ. (2021) 372:n71. 33782057 10.1136/bmj.n71 PMC8005924

[27] Li J Li C Puts M Wu YC Lyu MM Yuan B . Effectiveness of mindfulness-based interventions on anxiety, depression, and fatigue in people with lung cancer: A systematic review and meta-analysis. Int J Nurs Stud. (2023) 140:104447. doi: 10.1016/j.ijnurstu.2023.104447, PMID: 36796118

[28] Lo SB Holtze M Post KE Eche-Ugwu IJ Cooley ME Pirl WF . Depression and anxiety as moderators for a behavioral treatment for dyspnea in advanced lung cancer. J Pain Symptom Manage. (2025) 70:e121–e8. doi: 10.1016/j.jpainsymman.2025.03.030, PMID: 40188892 PMC12229764

[29] Hu H Yang W Liu Z Zhang X Shi J Xu H . Effect of eye movement training on sleep quality of patients with advanced lung cancer based on Pittsburgh sleep quality index. J Healthc Eng. (2021) 2021:9811980. doi: 10.1155/2021/9811980, PMID: 34956583 PMC8702321

[30] Quadflieg K Arents E Haesevoets S Van Hulle F Hermans F Criel M . Impact of cancer treatment on physical functioning, symptoms and health-related quality of life in patients with non-small cell lung cancer: A longitudinal observational study. Respir Med. (2025) 247:108283. doi: 10.1016/j.rmed.2025.108283, PMID: 40752628

[31] Zhu L Liu J Zeng L Moonindranath S An P Chen H . Thoracic high resolution computed tomography evaluation of imaging abnormalities of 108 lung cancer patients with different pulmonary function. Cancer Imaging. (2024) 24:78. doi: 10.1186/s40644-024-00720-9, PMID: 38910260 PMC11194896

[32] Jiang L Li K Lu S Hong Z Wang Y Xie Q . Mapping the EORTC QLQ-C30 and QLQ-LC13 to the SF-6D utility index in patients with lung cancer using machine learning and traditional regression methods. Health Qual Life Outcomes. (2025) 23:66. doi: 10.1186/s12955-025-02394-8, PMID: 40597342 PMC12220268

[33] Higgins JPT . Revised Cochrane risk of bias tool for randomized trials (RoB 2.0)(2016). Available online at: https://wwwriskofbiasinfo/welcome/rob-2-0-tool/archive-rob-2-0-2016.

[34] Cheung DST Takemura N Lam TC Ho JCM Deng W Smith R . Feasibility of aerobic exercise and Tai-Chi interventions in advanced lung cancer patients: A randomized controlled trial. Integr Cancer Ther. (2021) 20:15347354211033352. doi: 10.1177/15347354211033352, PMID: 34549648 PMC8461121

[35] Gu Y Wang Y Zhou H Qi A Wu G Li J . Efficacy of Chinese medicine on postoperative rehabilitation of non-small cell lung cancer (NSCLC), a randomized controlled study. Integr Cancer Ther. (2025) 24:15347354251314529. doi: 10.1177/15347354251314529, PMID: 39915957 PMC11803757

[36] Jiang M Zhao H Liu JH Zhao XW Jin LY Pan RJ . Does Tai Chi improve antioxidant and anti-inflammatory abilities via the KEAP1-NRF2 pathway and increase blood oxygen level in lung cancer patients: A randomized controlled trial? Eur J Integr Med. (2020) 37. doi: 10.1016/j.eujim.2020.101161

[37] Liu Y Liang X Yang B Wu Y Qian Y . Impact of Baduanjin Qigong exercise on fatigue in patients with lung cancer: A randomized controlled trial. J palliative Med. (2024) 27:1648–52. doi: 10.1089/jpm.2024.0194, PMID: 39585745

[38] Molassiotis A Vu DV Ching SSY . The effectiveness of qigong in managing a cluster of symptoms (Breathlessness-fatigue-anxiety) in patients with lung cancer: A randomized controlled trial. Integr Cancer Ther. (2021) 20:15347354211008253. doi: 10.1177/15347354211008253, PMID: 33847150 PMC8047940

[39] Wu J Zhang C Jing Z Wu X . Randomized controlled trial investigating the effect of a Baduanjin exercise plus nutrition programme on cancer-related fatigue in elderly lung cancer patients receiving chemotherapy. Exp Gerontol. (2025) 206:112763. doi: 10.1016/j.exger.2025.112763, PMID: 40305876

[40] Xu J Li X Zeng J Zhou Y Li Q Bai Z . Effect of Baduanjin qigong on postoperative pulmonary rehabilitation in patients with non-small cell lung cancer: a randomized controlled trial. Supportive Care Cancer. (2023) 32., PMID: 38158422 10.1007/s00520-023-08194-4

[41] Zhang YJ Wang R Chen PJ Yu DH . Effects of Tai Chi Chuan training on cellular immunity in post-surgical non-small cell lung cancer survivors: A randomized pilot trial. J Sport Health Science. (2013) 2:104–8. doi: 10.1016/j.jshs.2013.02.001

[42] Yu J Liu D Geng Y . Effects of five animal exercises combined with traditional Chinese medicine appropriate techniques based on syndrome differentiation on gastrointestinal and immune function in non-small cell lung cancer patients undergoing chemotherapy. Hebei J Traditional Chin Med. (2024) 46:821–4, 8.

[43] Guan Li Yang Z . Rehabilitative effects of Baduanjin combined with five-element music therapy on patients after lung cancer chemotherapy. J Chin Med Pharm. (2019) 25:102–4, 23.

[44] Liu S Xu J Yang J Yao F . Effects of Baduanjin health exercise on cancer-related fatigue and quality of life in lung cancer chemotherapy patients. Yunnan J Traditional Chin Med Pharm. (2021) 42:99–101.

[45] Ying Z Xie L Ji Yu Chen H . Application of stepwise Baduanjin exercise in the enhanced recovery of patients with non-small cell lung cancer brain metastases after surgery. Zhejiang J Traditional Chin Med. (2024) 59:704–6.

[46] Zhang S Liao X Hong Y Nie L Peng Z . Effects of Baduanjin training on health fitness and quality of life in non-small cell lung cancer patients undergoing radiotherapy. Zhejiang Clin Med. (2024) 26:1496–8.

[47] Zhang Q Xu G . Effects of six-word formula breathing training on respiratory function and enhanced recovery in patients after thoracoscopic lung cancer surgery. Chin J Health Preservation. (2024) 42:66–8, 75.

[48] Li Q Wang L Jiao H . Application of Baduanjin in the rehabilitation of patients after non-small cell lung cancer surgery. Nurs Res. (2017) 31:3755–9.

[49] Yang C Zhang Q . A study on the effect of personalized psychological nursing combined with “Six-word formula” Breathing exercises in improving anxiety, depression, and quality of life in patients after lung cancer surgery. J Traditional Chin Med External Ther. (2021) 30:76–8.

[50] Wang J Li Z Ren X Xian Q Guo H Teng Y . Study on the role of six-character formula respiratory function exercise in the rehabilitation of lung cancer surgery patients. Contemp Nurse (Mid-Month Edition). (2022) 29:122–5.

[51] Wang L Lü H . Effects of Ba Duan Jin exercise intervention combined with pulmonary rehabilitation training on pulmonary function and SF-36 scores in patients after non-small cell lung cancer surgery. Electronic J Clin Med Literature. (2022) 9:1–3, 10.

[52] Su H Bi H . Effects of six-word formula intervention on cancer-related fatigue, pulmonary function, and exercise endurance in lung cancer patients. J Traditional Chin Med Shandong. (2024) 43:729–34, 50.

[53] Zhao N Liu D Zhang J . Effects of seated Baduanjin combined with specific respiratory training on pulmonary function and exercise endurance in lung cancer patients undergoing postoperative chemotherapy. Hebei J Traditional Chin Med. (2024) 46:628–32.

[54] Chen Y Guan H . Effects of bird exercise from the five animal exercises on traditional Chinese medicine symptoms, exercise tolerance, and quality of life in lung cancer patients. Nurs Res. (2019) 33:4029–32.

[55] Han R Lin H . Clinical study on the efficacy of Ba Duan Jin Qigong in improving pulmonary function and quality of life in postoperative non-small cell lung cancer patients. Tianjin J Traditional Chin Med. (2016) 33:715–8.

[56] Duan T Guo Y Lu Q Pan H . Effect of pulmonary rehabilitation training on postoperative recovery in lung cancer patients undergoing thoracoscopic partial pulmonary resection: a meta-analysis. Am J Transl Res. (2024) 16:6168–86. doi: 10.62347/NJRM6592, PMID: 39678604 PMC11645604

[57] Chin MS Kales SN . Understanding mind-body disciplines: A pilot study of paced breathing and dynamic muscle contraction on autonomic nervous system reactivity. Stress Health. (2019) 35:542–8. doi: 10.1002/smi.2887, PMID: 31347763 PMC8758201

[58] Zhou SC Chen G Xu XY Zhang C Chen GM Chan YT . Comparative efficacy of various exercise types on cancer-related fatigue for cancer survivors: A systematic review and network meta-analysis of randomized controlled trials. Cancer Med. (2025) 14. doi: 10.1002/cam4.70816, PMID: 40145635 PMC11948276

[59] Yan ZL Chen M Tao JJ Wang YC Huang P . Effectiveness of Baduanjin exercise on pulmonary function, quality of life, psychological well-being and exercise tolerance in postoperative patients with non-small cell lung cancer: Systematic review and meta-analysis of randomized controlled trials. Explore-the J Sci Healing. (2025) 21. doi: 10.1016/j.explore.2025.103186, PMID: 40382884

[60] Lo WY Liu XH Cheung DST Lin CC . Effects of Tai Chi and Qigong in depressive and anxiety symptoms in cancer patients: an overview of systematic reviews. Heart Mind. (2025) 9:136–46. doi: 10.4103/hm.HM-D-24-00090

[61] Kayser J Hu RX Rosenscruggs D Li L Xiang XL . A systematic review of the impact of select mindfulness interventions on psychological outcomes among older adults with chronic health conditions. Clin Gerontologist. (2023) 46:302–14. doi: 10.1080/07317115.2022.2076636, PMID: 35585039

[62] Singh B Olds T Curtis R Dumuid D Virgara R Watson A . Effectiveness of physical activity interventions for improving depression, anxiety and distress: an overview of systematic reviews. Br J Sports Med. (2023) 57. doi: 10.1136/bjsports-2022-106195, PMID: 36796860 PMC10579187

[63] Hasan F Tu YK Lin CM Chuang LP Jeng C Yuliana LT . Comparative efficacy of exercise regimens on sleep quality in older adults: A systematic review and network meta-analysis. Sleep Med Rev. (2022) 65. doi: 10.1016/j.smrv.2022.101673, PMID: 36087457

[64] Yang GY Hunter J Bu FL Hao WL Zhang H Wayne PM . Determining the safety and effectiveness of Tai Chi: a critical overview of 210 systematic reviews of controlled clinical trials. Systematic Rev. (2022) 11. doi: 10.1186/s13643-022-02100-5, PMID: 36463306 PMC9719113

