# Supplementary material for: The impact of traditional mind–body exercises on pulmonary function, exercise capacity, and quality of life in patients with lung cancer: a systematic review and meta-analysis
Source: Front Oncol. 2025 Dec 9;15:1716583. doi: 10.3389/fonc.2025.1716583 (PMC12722919; doi:10.3389/fonc.2025.1716583)
Supplement: Supplementary file 1 [file DataSheet1.docx]

Table s1 search strategy

| Database | search strategy | results |
| --- | --- | --- |
| PubMed | (("Mind-Body Therapies"[Mesh]) OR ((((((((((((((((((((Mind-Body Therapies[Title/Abstract]) OR (Tai Ji[Title/Abstract])) OR (Tai-ji[Title/Abstract])) OR (Tai Chi[Title/Abstract])) OR (Chi, Tai[Title/Abstract])) OR (Tai Chi Chuan[Title/Abstract])) OR (Taiji[Title/Abstract])) OR (Taijiquan[Title/Abstract])) OR (T'ai Chi[Title/Abstract])) OR (Tai Ji Quan[Title/Abstract])) OR (Ji Quan, Tai[Title/Abstract])) OR (Quan, Tai Ji[Title/Abstract])) OR (Eight section brocade[Title/Abstract])) OR (Baduanjin[Title/Abstract])) OR (Wuqinxi[Title/Abstract])) OR (Yijinjing[Title/Abstract])) OR (Liuzijue[Title/Abstract])) OR (Qigong[Title/Abstract])) OR (Five elements boxing[Title/Abstract])) OR (Wuqinxi[Title/Abstract]))) AND (("Lung Neoplasms"[Mesh]) OR ((((((((((((((((((Lung Neoplasms[Title/Abstract]) OR (Neoplasms, Pulmonary[Title/Abstract])) OR (Neoplasm, Pulmonary[Title/Abstract])) OR (Pulmonary Neoplasm[Title/Abstract])) OR (Pulmonary Neoplasms[Title/Abstract])) OR (Neoplasms, Lung[Title/Abstract])) OR (Lung Neoplasm[Title/Abstract])) OR (Neoplasm, Lung[Title/Abstract])) OR (Lung Cancer[Title/Abstract])) OR (Cancer, Lung[Title/Abstract])) OR (Cancers, Lung[Title/Abstract])) OR (Lung Cancers[Title/Abstract])) OR (Cancer of Lung[Title/Abstract])) OR (Pulmonary Cancer[Title/Abstract])) OR (Cancer, Pulmonary[Title/Abstract])) OR (Cancers, Pulmonary[Title/Abstract])) OR (Pulmonary Cancers[Title/Abstract])) OR (Cancer of the Lung[Title/Abstract]))) | 169 |
| Embase | \| #45 \| #40 AND #44 \| 36 \| \| --- \| --- \| --- \| \| #44 \| #41 OR #42 OR #43 \| 1058455 \| \| #43 \| 'double-blind':ab,ti \| 295258 \| \| #42 \| 'placebo':ab,ti \| 463313 \| \| #41 \| 'random':ab,ti \| 521104 \| \| #40 \| #20 AND #39 \| 1165 \| \| #39 \| #21 OR #22 OR #23 OR #24 OR #25 OR #26 OR #27 OR #28 OR #29 OR #30 OR #31 OR #32 OR #33 OR #34 OR #35 OR #36 OR #37 OR #38 \| 676685 \| \| #38 \| 'cancer of the lung':ab,ti \| 2291 \| \| #37 \| 'pulmonary cancers':ab,ti \| 273 \| \| #36 \| 'cancers, pulmonary':ab,ti \| 48 \| \| #35 \| 'cancer, pulmonary':ab,ti \| 576 \| \| #34 \| 'pulmonary cancer':ab,ti \| 1457 \| \| #33 \| 'cancer of lung':ab,ti \| 151 \| \| #32 \| 'lung cancers':ab,ti \| 24647 \| \| #31 \| 'cancers, lung':ab,ti \| 613 \| \| #30 \| 'cancer, lung':ab,ti \| 4121 \| \| #29 \| 'lung cancer':ab,ti \| 360018 \| \| #28 \| 'neoplasm, lung':ab,ti \| 26 \| \| #27 \| 'lung neoplasm':ab,ti \| 598 \| \| #26 \| 'neoplasms, lung':ab,ti \| 61 \| \| #25 \| 'pulmonary neoplasms':ab,ti \| 761 \| \| #24 \| 'pulmonary neoplasm':ab,ti \| 477 \| \| #23 \| 'neoplasms, pulmonary':ab,ti \| 29 \| \| #22 \| 'lung neoplasms':ab,ti \| 1183 \| \| #21 \| 'lung tumor'/exp \| 642589 \| \| #20 \| #1 OR #2 OR #3 OR #4 OR #5 OR #6 OR #7 OR #8 OR #9 OR #10 OR #11 OR #12 OR #13 OR #14 OR #15 OR #16 OR #17 OR #18 OR #19 \| 99634 \| \| #19 \| 'wuqinxi':ab,ti \| 74 \| \| #18 \| 'five elements boxing':ab,ti \| 0 \| \| #17 \| 'qigong':ab,ti \| 1787 \| \| #16 \| 'liuzijue':ab,ti \| 71 \| \| #15 \| 'yijinjing':ab,ti \| 65 \| \| #14 \| 'wuqinxi':ab,ti \| 74 \| \| #13 \| 'baduanjin':ab,ti \| 509 \| \| #12 \| 'eight section brocade':ab,ti \| 20 \| \| #11 \| 'quan, tai ji':ab,ti \| 2 \| \| #10 \| 'ji quan, tai':ab,ti \| 2 \| \| #9 \| 'tai chi':ab,ti \| 4102 \| \| #8 \| 'taijiquan':ab,ti \| 122 \| \| #7 \| 'taiji':ab,ti \| 188 \| \| #6 \| 'tai chi chuan':ab,ti \| 318 \| \| #5 \| 'chi, tai':ab,ti \| 18 \| \| #4 \| 'tai chi':ab,ti \| 4102 \| \| #3 \| 'tai ji':ab,ti \| 110 \| \| #2 \| 'alternative medicine':ab,ti \| 16166 \| \| #1 \| 'alternative medicine'/exp \| 90354 \| | 36 |
| Web of science | \|  \|  \|  \| \| --- \| --- \| --- \| \| 1 \| TS=(Mind-Body Therapies) OR TS=(Tai Ji) OR TS=(Tai-ji) OR TS=(Tai Chi) OR TS=(Chi, Tai) OR TS=(Tai Chi Chuan) OR TS=(Taiji) OR TS=(Taijiquan) OR TS=(T'ai Chi) OR TS=(Tai Ji Quan) OR TS=(Ji Quan, Tai) OR TS=(Quan, Tai Ji) OR TS=(Eight section brocade) OR TS=(Baduanjin) OR TS=(Wuqinxi ) OR TS=(Yijinjing) OR TS=(Liuzijue) OR TS=(Qigong ) OR TS=(Five elements boxing) OR TS=(Wuqinxi) \| 10210 \| \| 2 \| TS=(Lung Neoplasms) OR TS=(Neoplasms, Pulmonary) OR TS=(Neoplasm, Pulmonary) OR TS=(Pulmonary Neoplasm) OR TS=(Pulmonary Neoplasms) OR TS=(Neoplasms, Lung) OR TS=(Lung Neoplasm) OR TS=(Neoplasm, Lung) OR TS=(Lung Cancer) OR TS=(Cancer, Lung) OR TS=(Cancers, Lung) OR TS=(Lung Cancers) OR TS=(Cancer of Lung) OR TS=(Pulmonary Cancer) OR TS=(Cancer, Pulmonary) OR TS=(Cancers, Pulmonary) OR TS=(Pulmonary Cancers) OR TS=(Cancer of the Lung) \| 507914 \| \| 3 \| #1 AND #2 \| 132 \| | 132 |
| Cochrane | #1 MeSH descriptor: [Mind-Body Therapies] explode all trees 9820  #2 (Mind-Body Therapies):ti,ab,kw OR (Tai Ji):ti,ab,kw OR (Tai-ji):ti,ab,kw OR (Tai Chi):ti,ab,kw OR (Chi, Tai):ti,ab,kw 2474  #3 (Tai Chi Chuan):ti,ab,kw OR (Taiji):ti,ab,kw OR (Taijiquan):ti,ab,kw OR (T'ai Chi):ti,ab,kw OR (Tai Ji Quan):ti,ab,kw 450  #4 (Ji Quan, Tai):ti,ab,kw OR (Quan, Tai Ji):ti,ab,kw AND (Eight section brocade):ti,ab,kw OR (Baduanjin):ti,ab,kw OR (Wuqinxi):ti,ab,kw 591  #5 (Yijinjing):ti,ab,kw OR (Liuzijue):ti,ab,kw OR (Qigong):ti,ab,kw OR (Five elements boxing):ti,ab,kw OR (Wuqinxi):ti,ab,kw 999  #6 #1or#2or#3or#4or#5 12480  #7 MeSH descriptor: [Lung Neoplasms] explode all trees 12481  #8 (Lung Neoplasms):ti,ab,kw OR (Neoplasms, Pulmonary):ti,ab,kw OR (Neoplasm, Pulmonary):ti,ab,kw OR (Pulmonary Neoplasm):ti,ab,kw OR (Pulmonary Neoplasms):ti,ab,kw 16250  #9 (Neoplasms, Lung):ti,ab,kw OR (Lung Neoplasm):ti,ab,kw OR (Neoplasm, Lung):ti,ab,kw OR (Lung Cancer):ti,ab,kw OR (Cancer, Lung):ti,ab,kw 35508  #10 (Cancers, Lung):ti,ab,kw OR (Lung Cancers):ti,ab,kw OR (Cancer of Lung):ti,ab,kw OR (Pulmonary Cancer):ti,ab,kw OR (Cancer, Pulmonary):ti,ab,kw 34110  #11 (Cancers, Pulmonary):ti,ab,kw OR (Pulmonary Cancers):ti,ab,kw OR (Cancer of the Lung):ti,ab,kw 30962  #12 #7or#8or#9or#10or#11 37637  #13 #6and#12 133 | 133 |
| CNKI | 主题（太极 + 八段锦 +身心疗法 +五行拳 + 五禽戏 + 六字诀 + 气功） AND主题(肺癌 + 肺肿瘤) | 44 |
| Wan fang | 主题（太极 OR 八段锦 OR身心疗法 OR五行拳 OR 五禽戏 OR 六字诀 气功） AND主题(肺癌 OR 肺肿瘤) | 116 |
| VIP. | 主题（太极 + 八段锦 +身心疗法 +五行拳 + 五禽戏 + 六字诀 + 气功） AND主题(肺癌 + 肺肿瘤) | 62 |
| CBM | 主题（太极 OR 八段锦 OR身心疗法 OR五行拳 OR 五禽戏 OR 六字诀 气功） AND主题(肺癌 OR 肺肿瘤) | 102 |


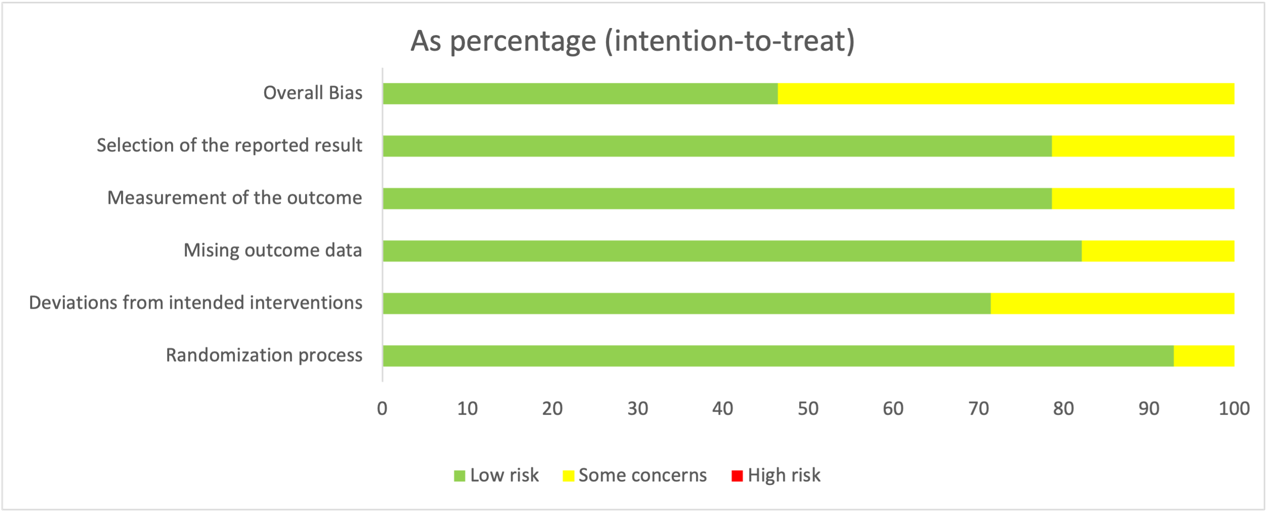


Figure S1 Risk bias of summary


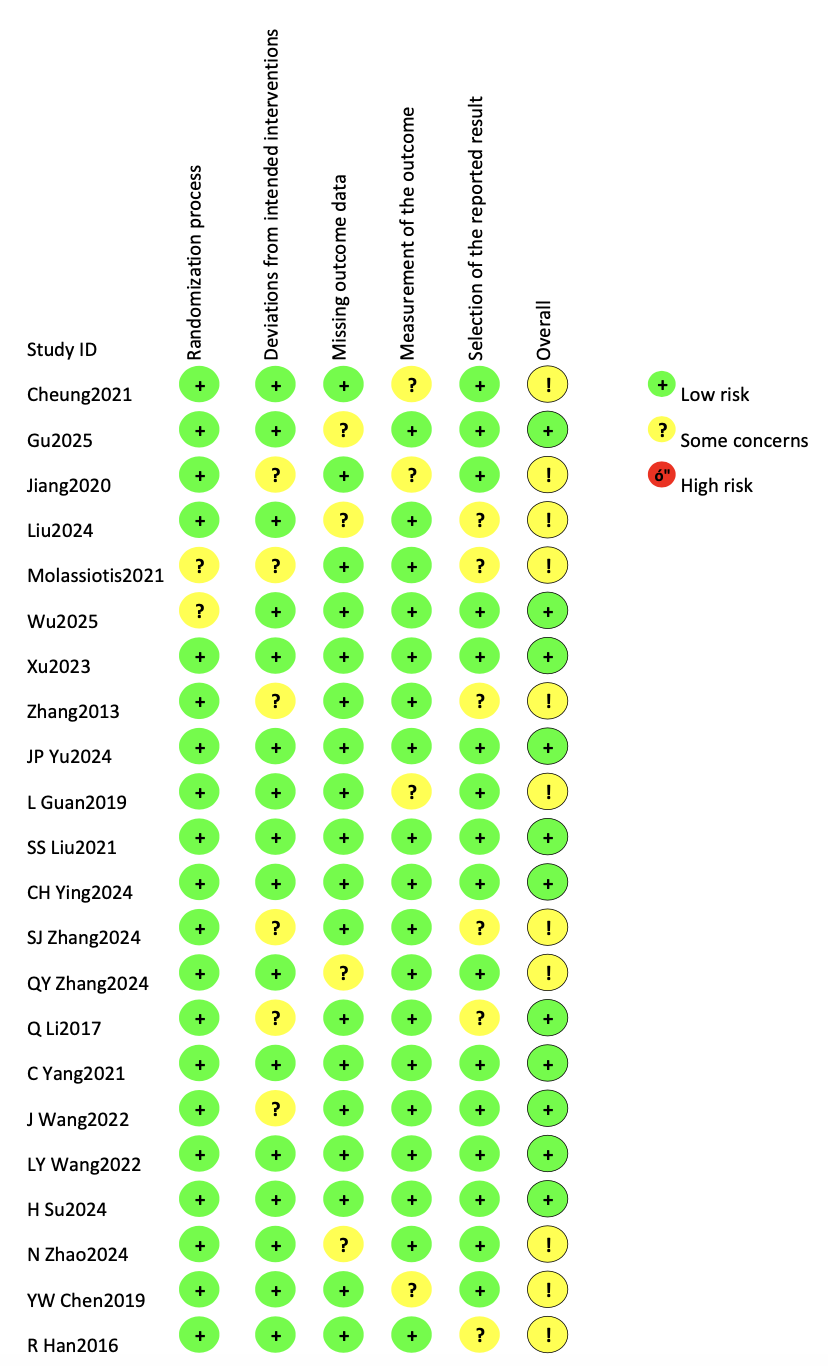


Figure S2 Risk bias of graph


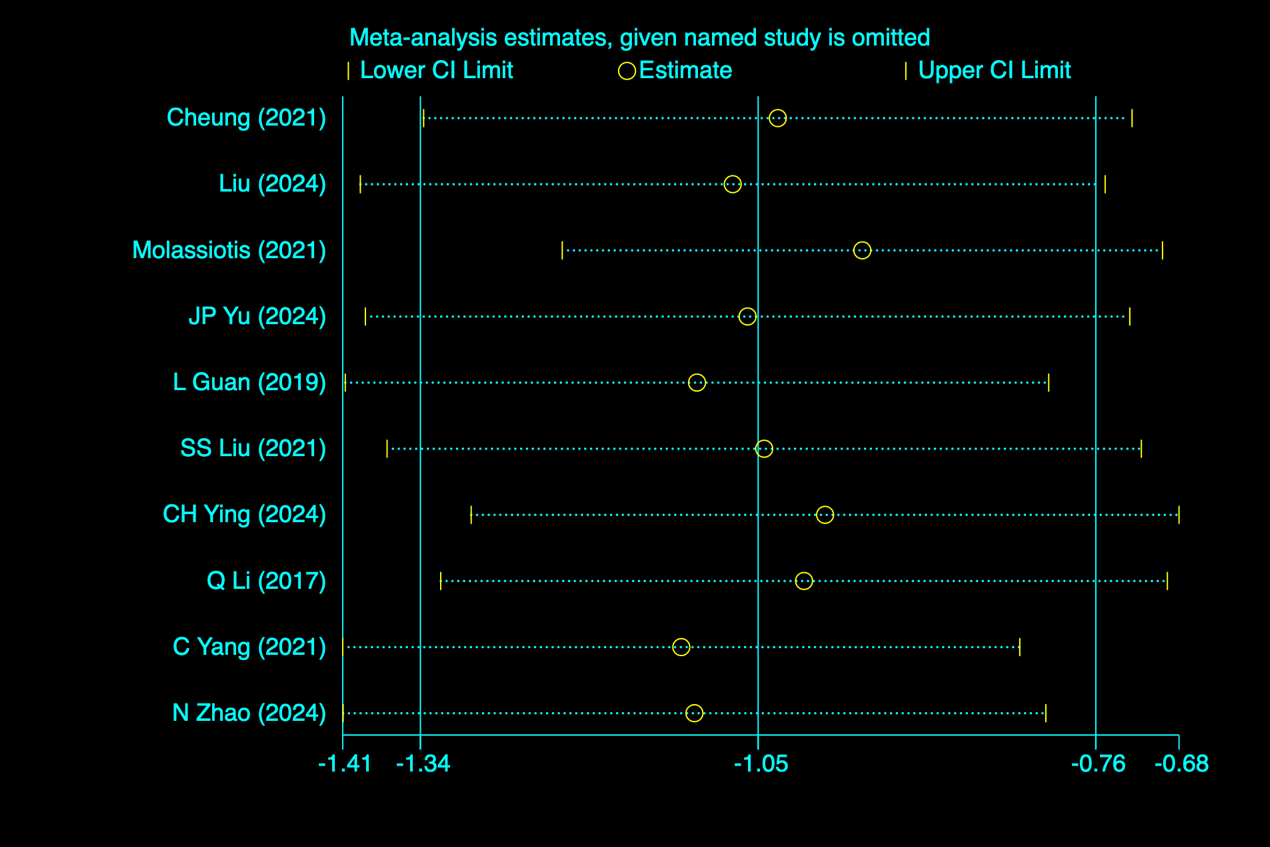


Figure S3 The sensitivity analysis results of the meta-analysis of anxiety scores


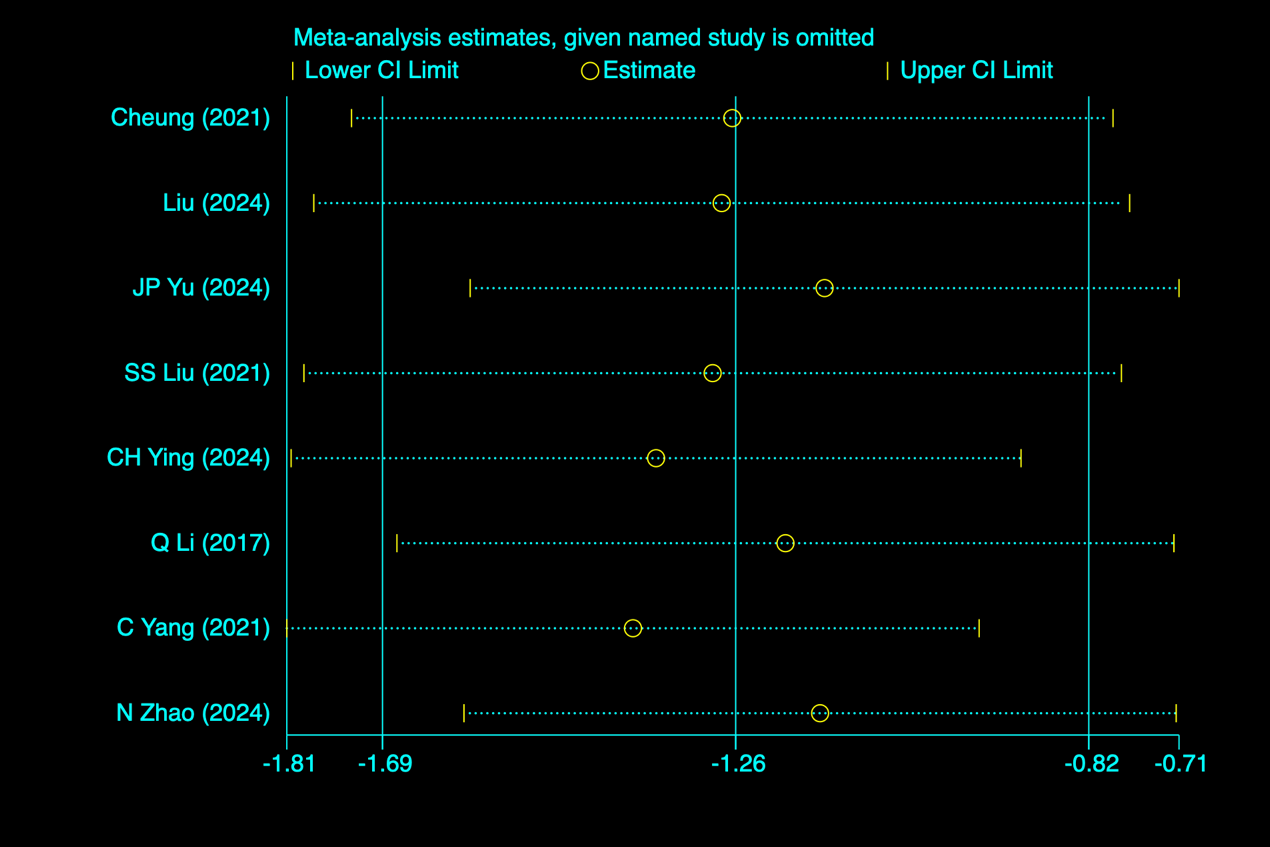


Figure S4 The sensitivity analysis results of the meta-analysis of depression scores


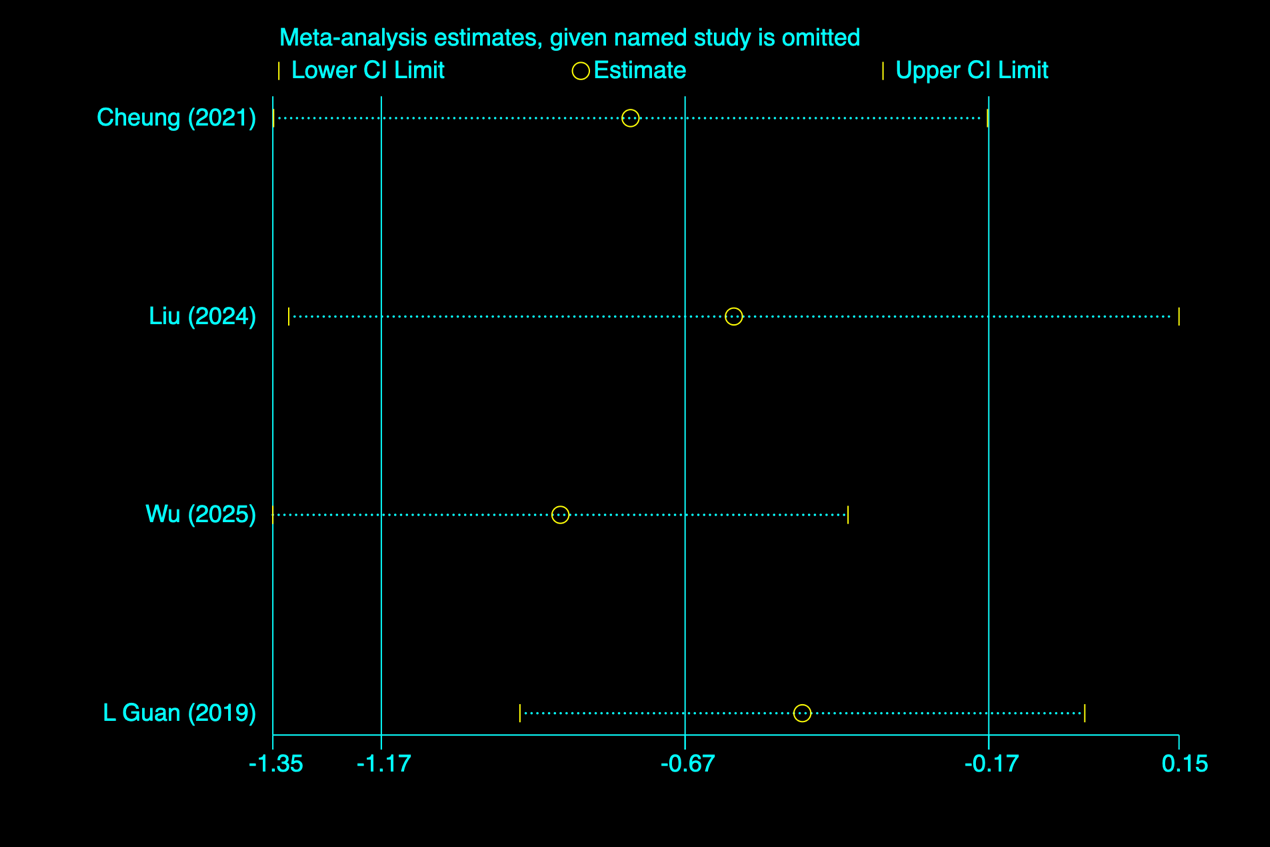


Figure S5 The sensitivity analysis results of the meta-analysis of PSQI scores


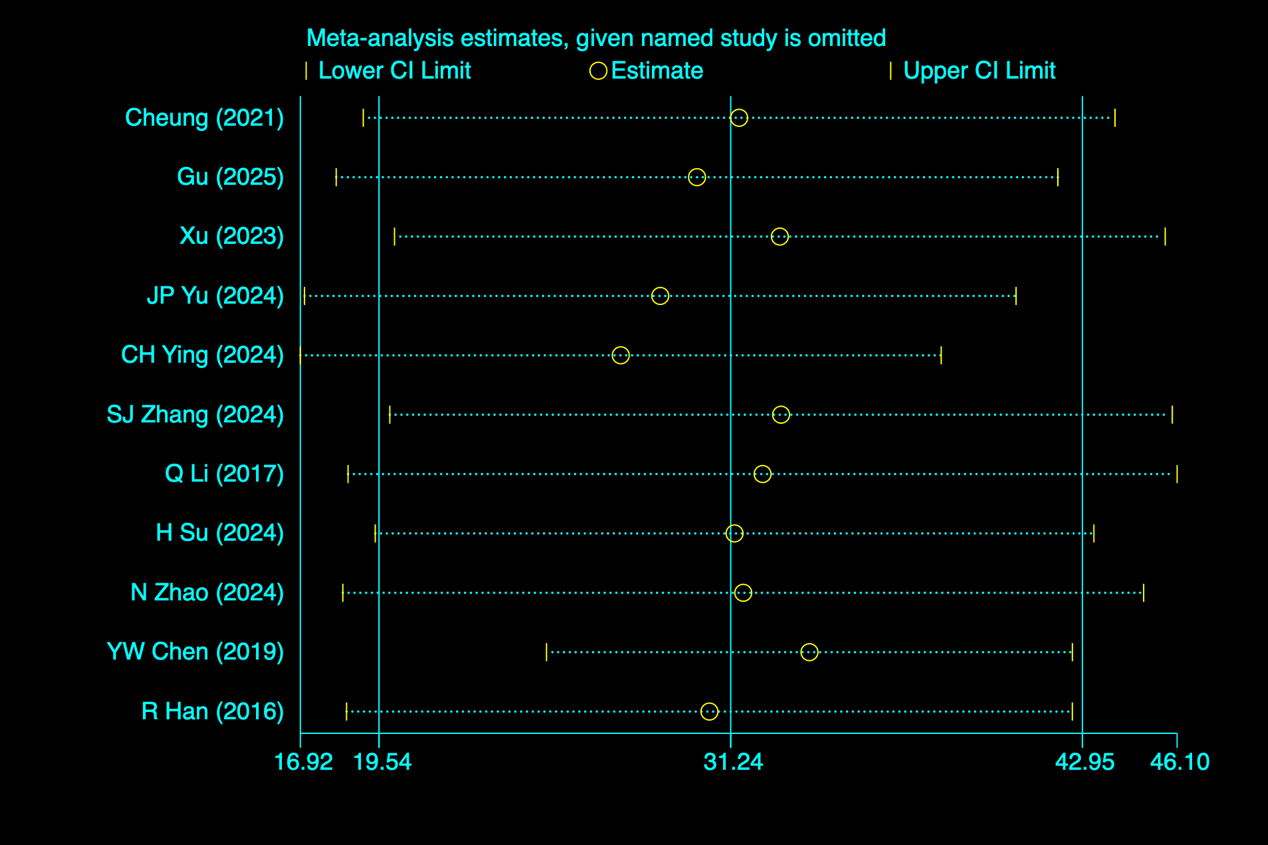


Figure S6 The sensitivity analysis results of the meta-analysis of 6MWT


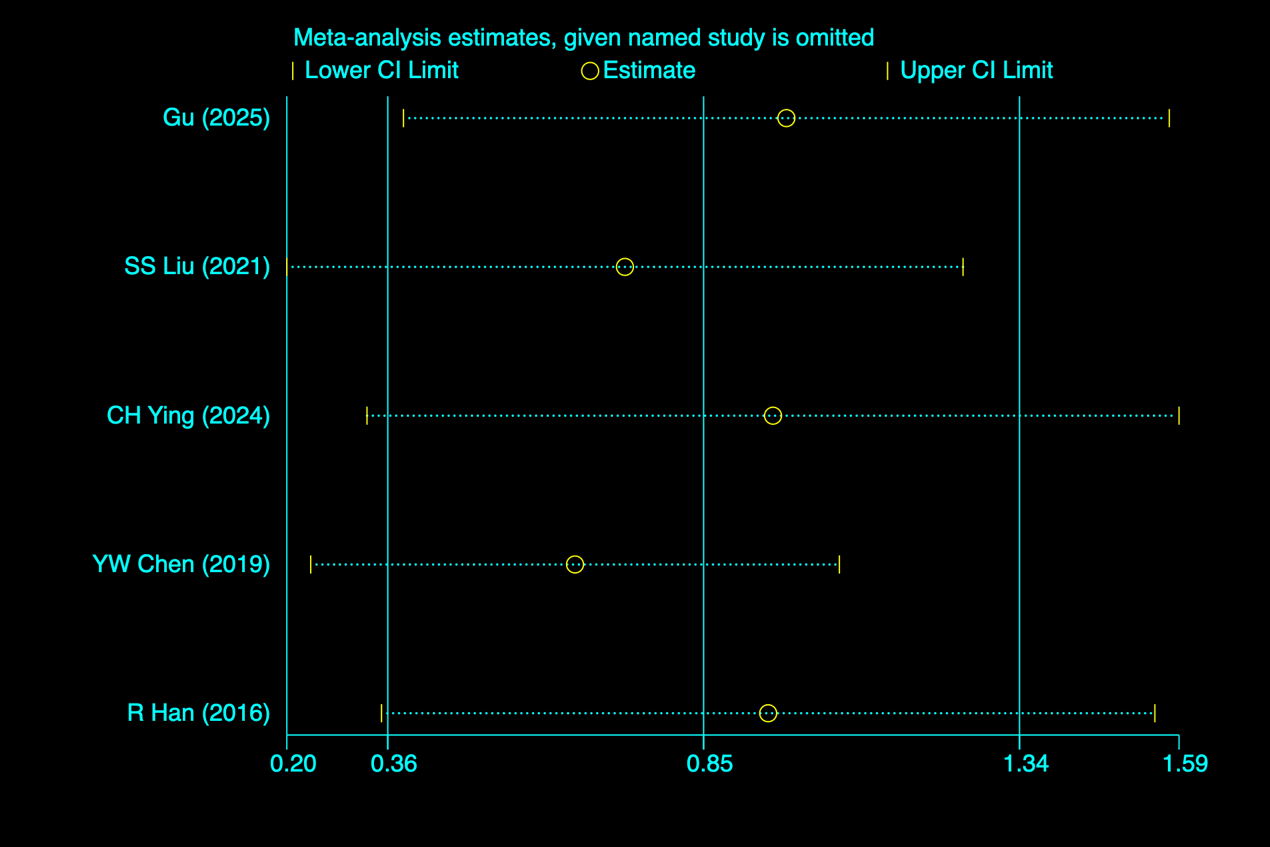


Figure S7 The sensitivity analysis results of the meta-analysis of quality of Life


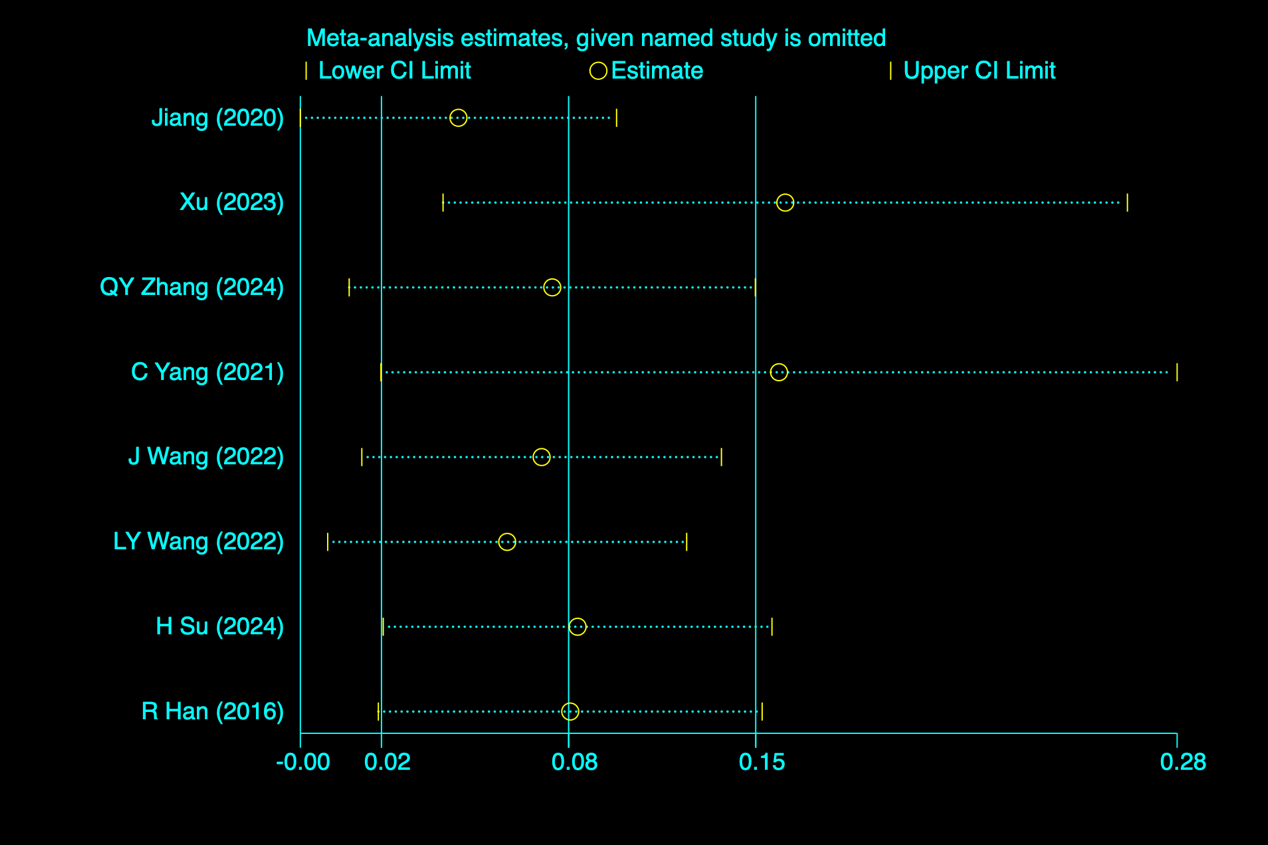


Figure S7 The sensitivity analysis results of the meta-analysis of FVC


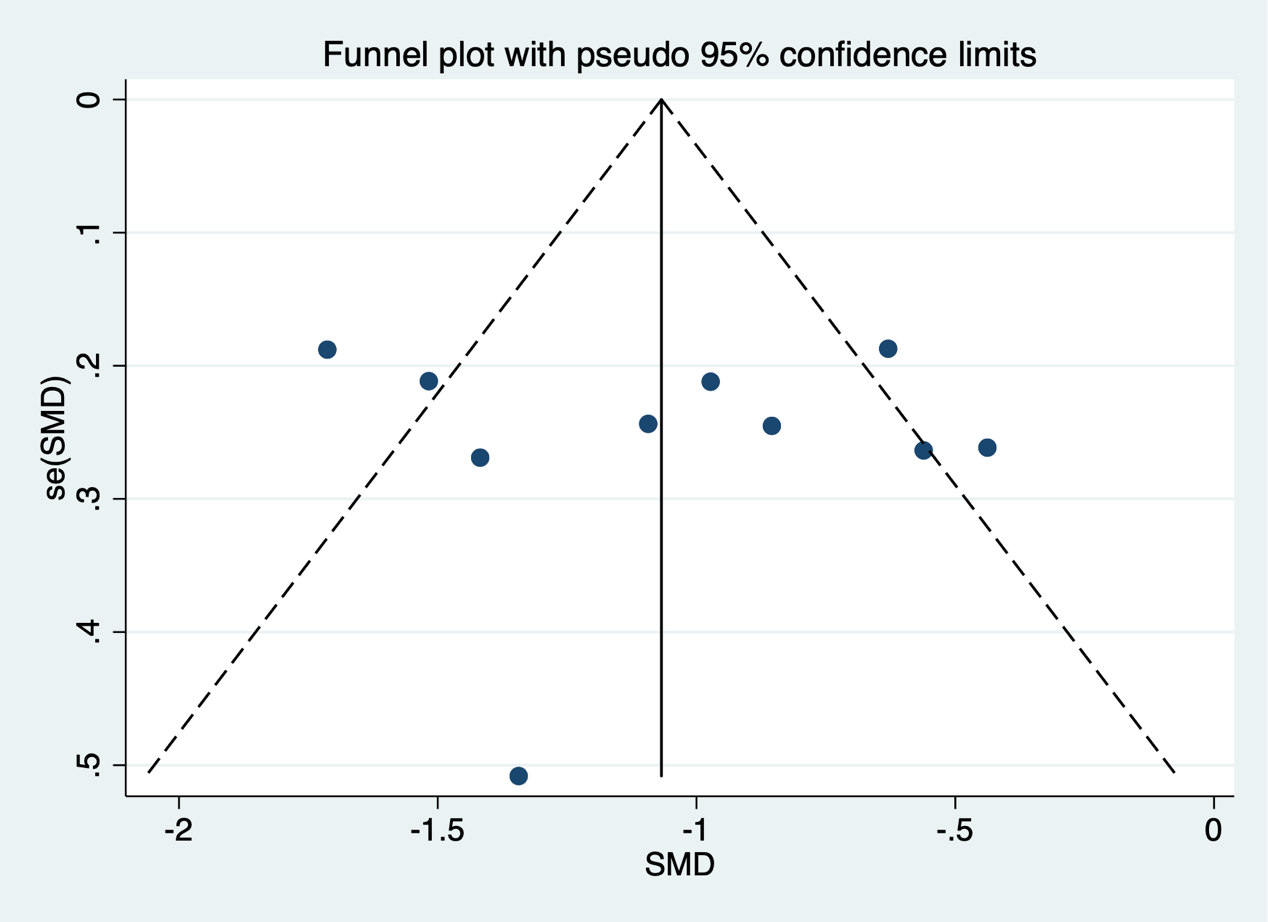


Figure S8 Meta-analysis funnel plot of anxiety score.


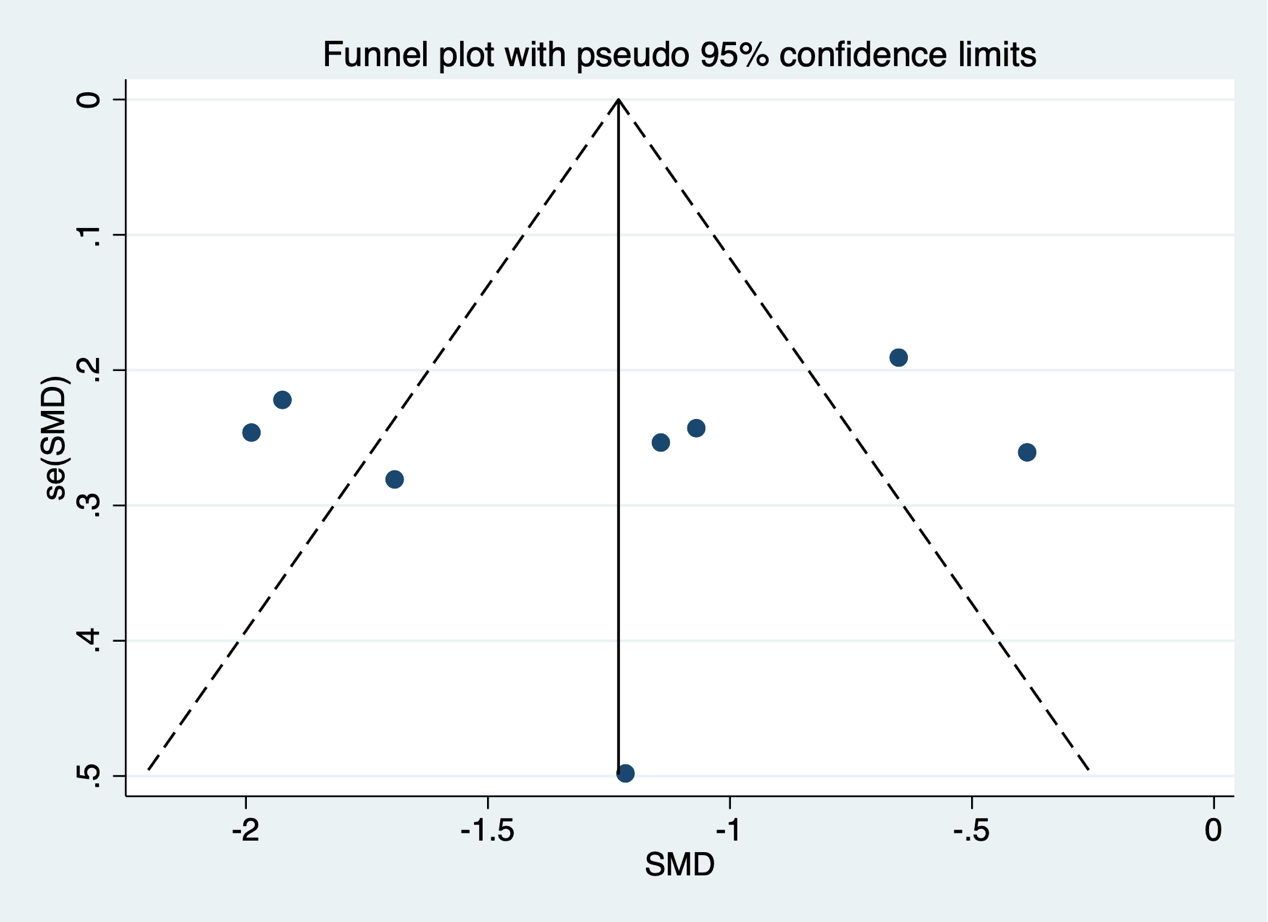


Figure S9 Meta-analysis funnel plot of depression score.


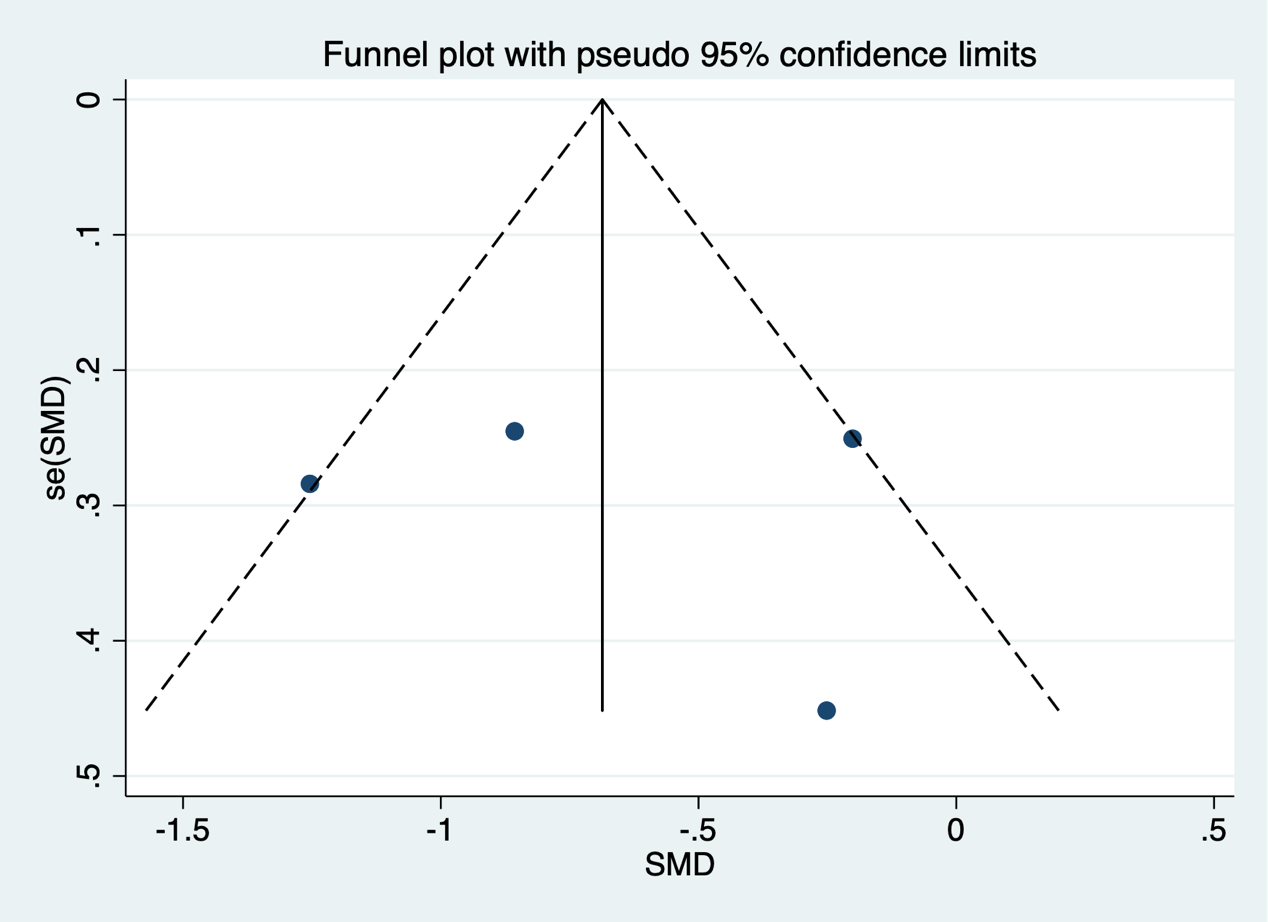


Figure S10 Meta-analysis funnel plot of PSQI score.


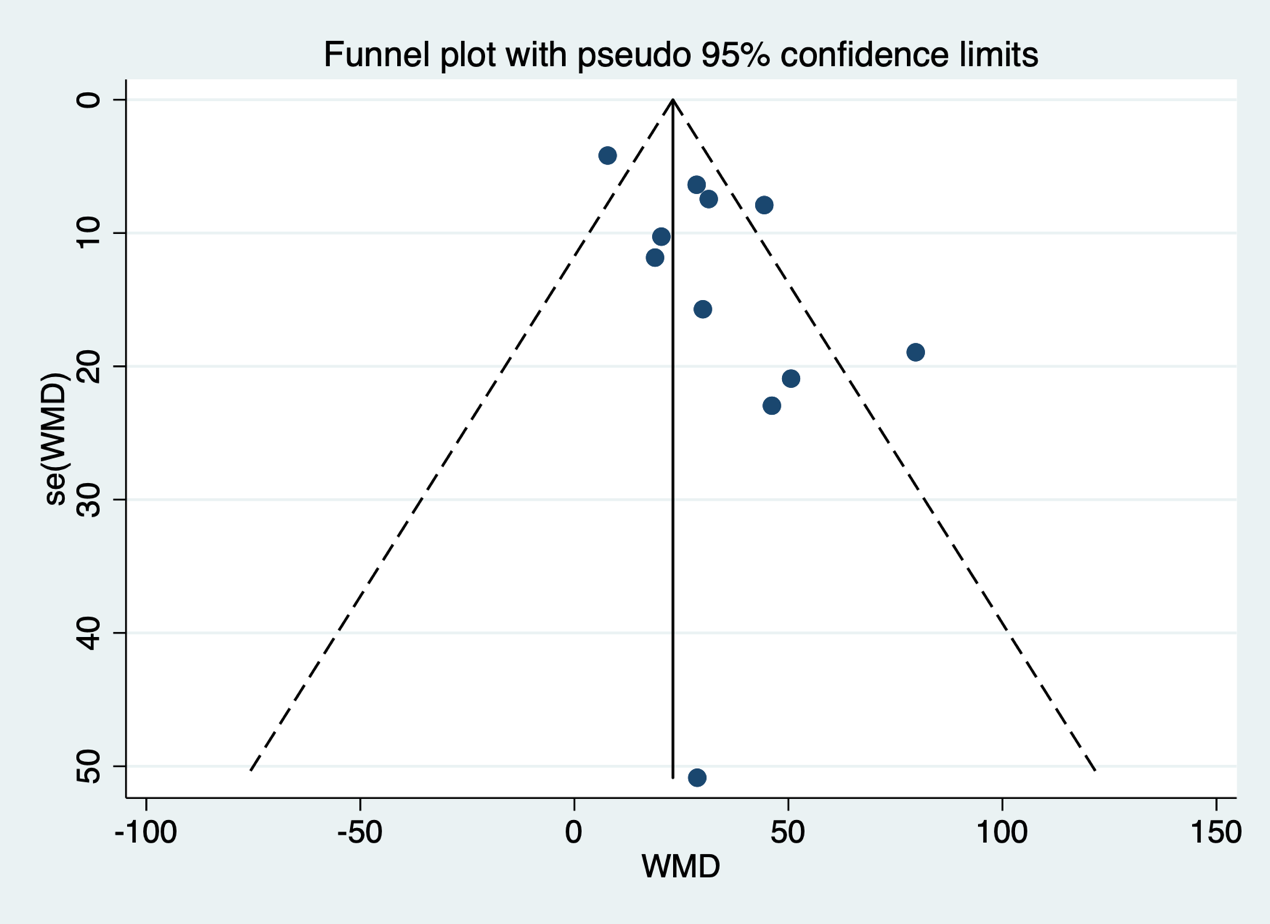


Figure S11 Meta-analysis funnel plot of 6MWT


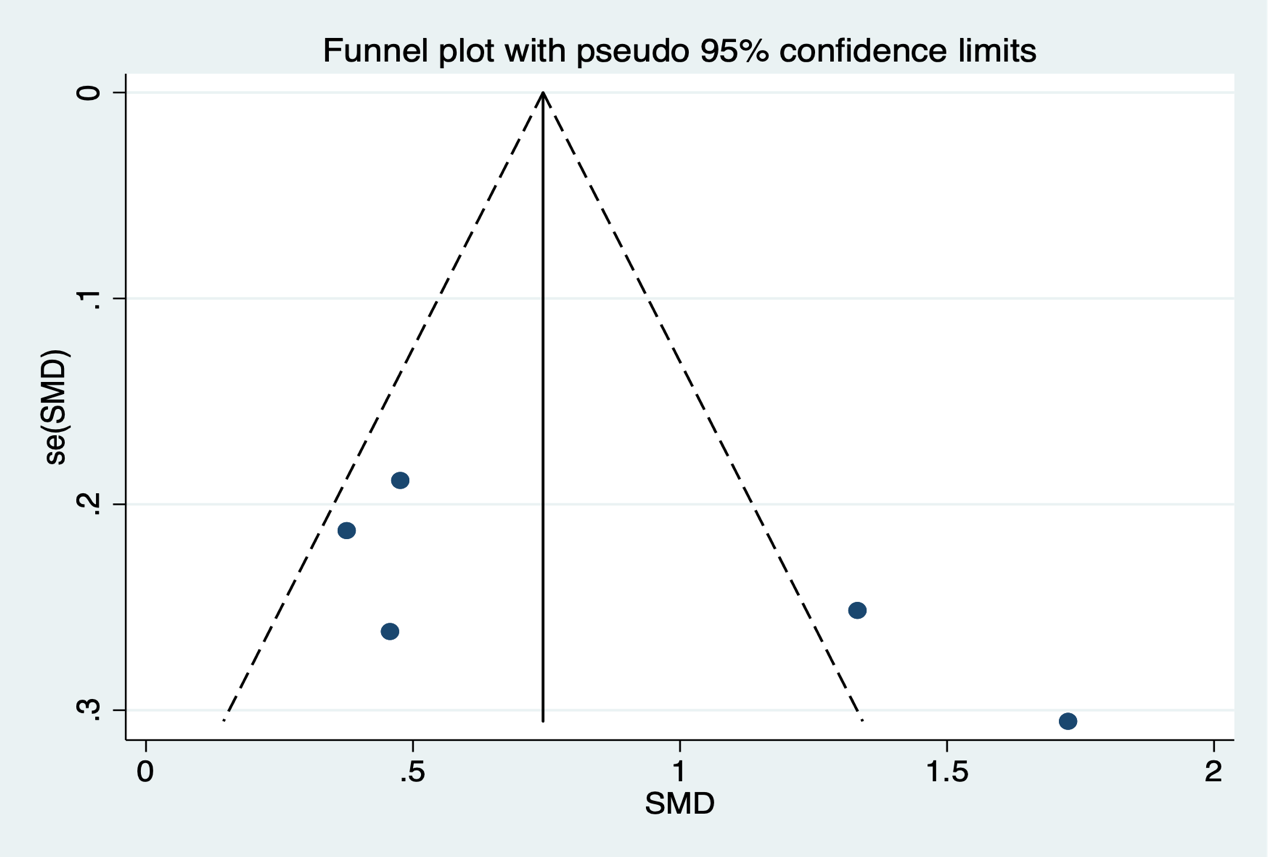


Figure S12 Meta-analysis funnel plot of quality of Life


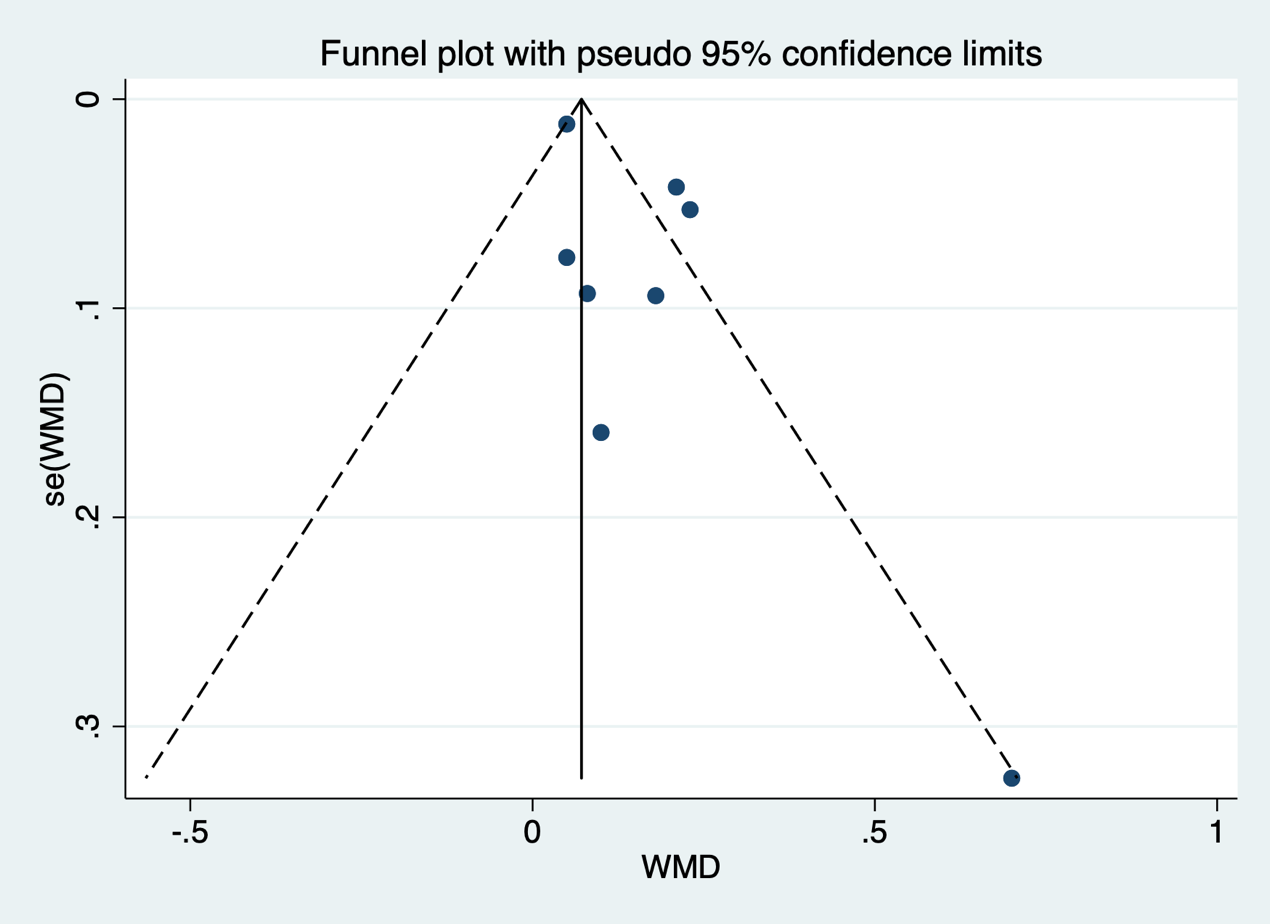


Figure S13 Meta-analysis funnel plot of FEV1


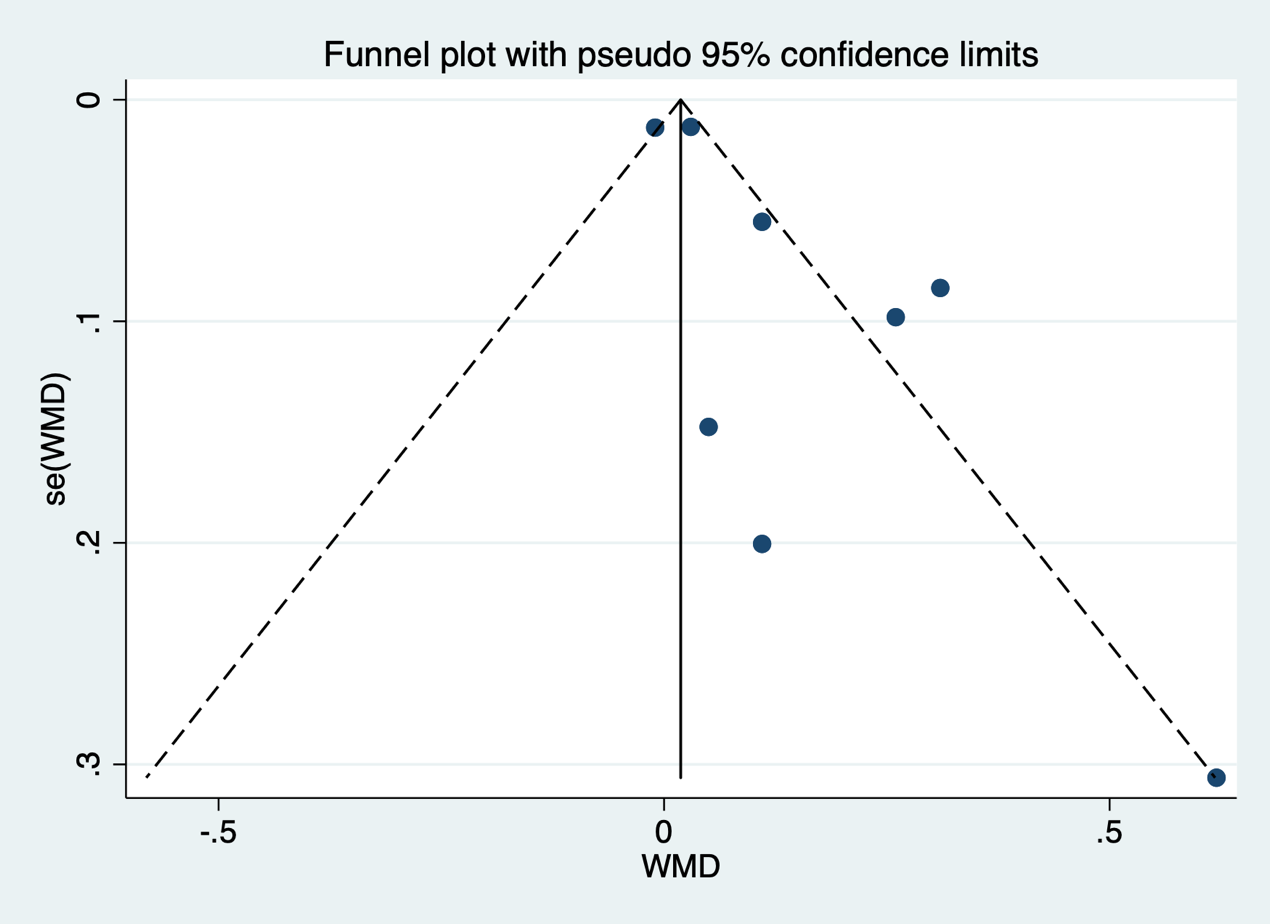


Figure S14 Meta-analysis funnel plot of FVC


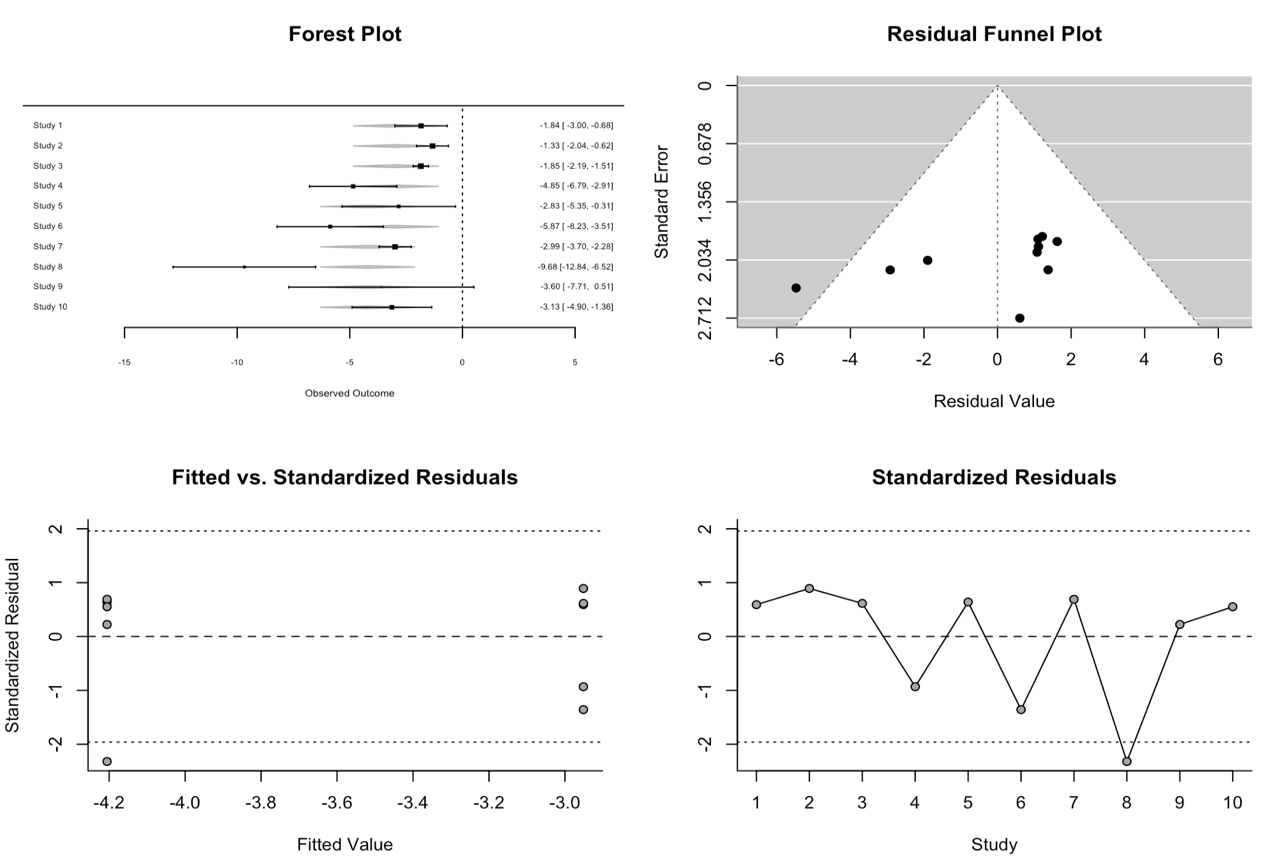


Figure S15 Figure of Meta regression analysis of Anxiety scores


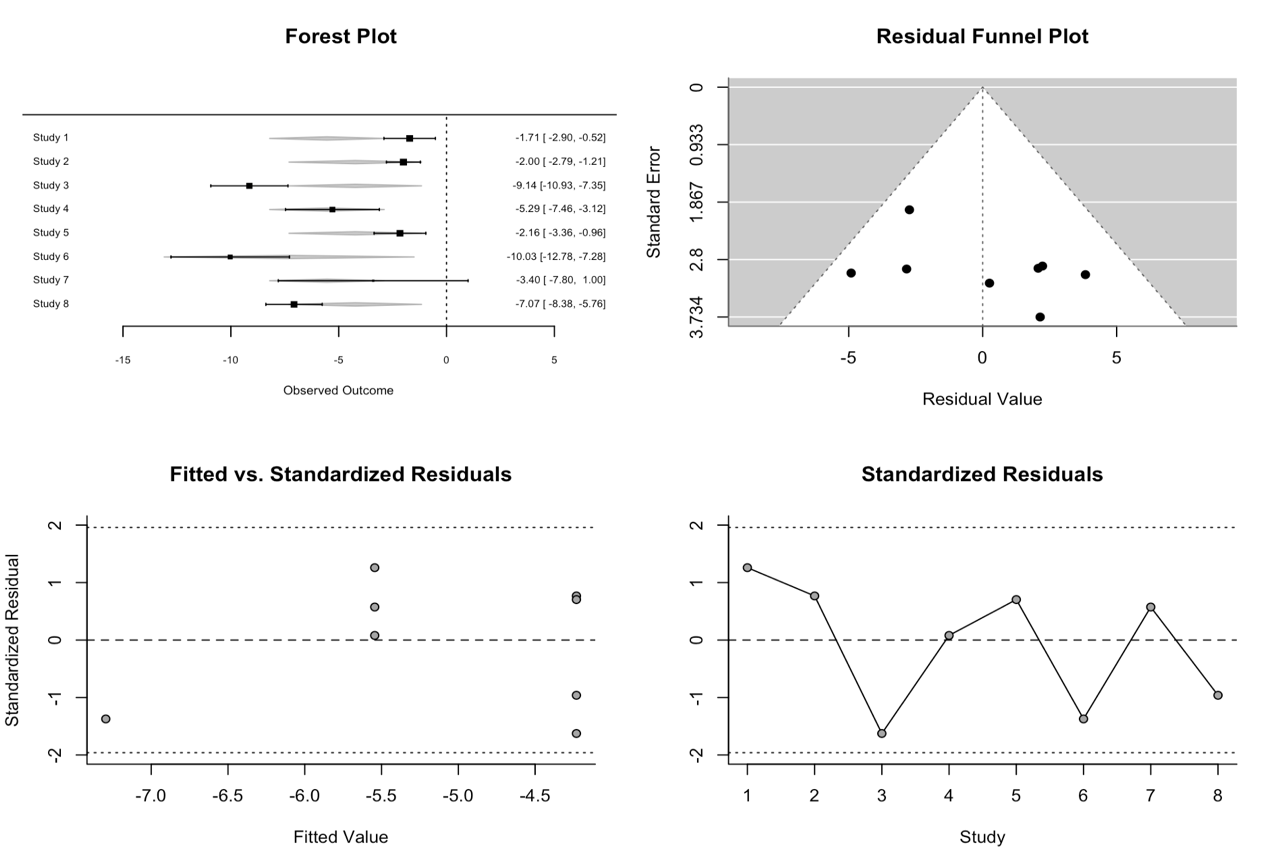


Figure S16 Figure of Meta regression analysis of Depression score


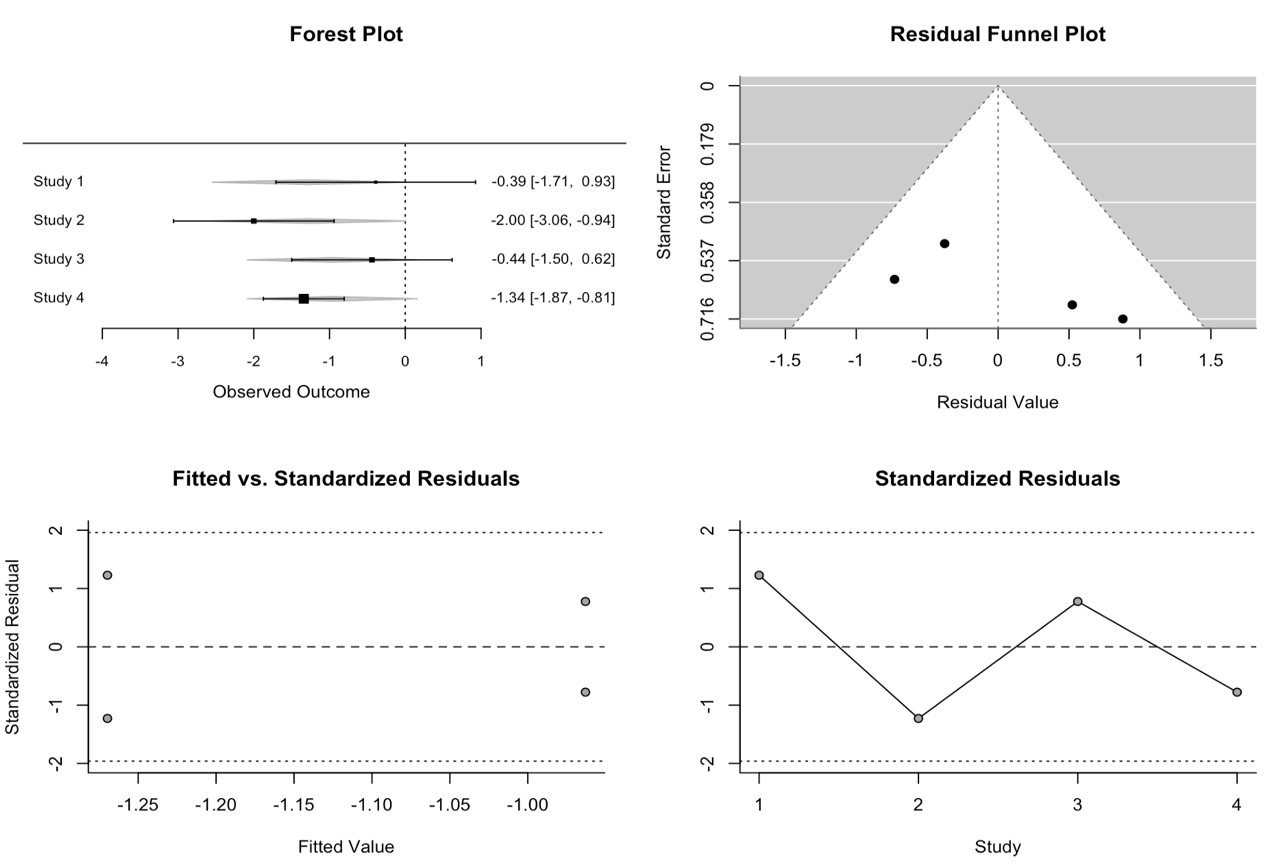


Figure S17 Figure of Meta regression analysis of PSQI


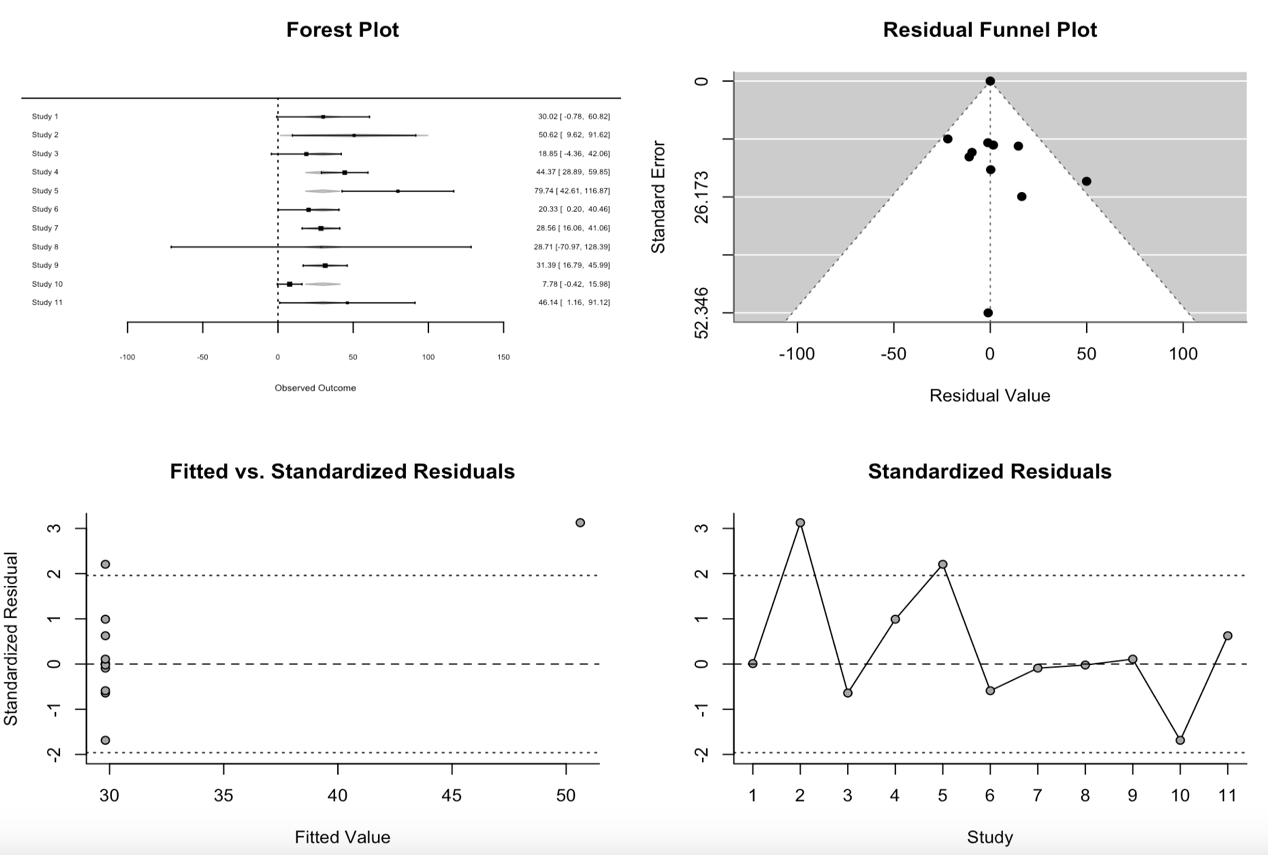


Figure S18 Figure of Meta regression analysis of 6MWT


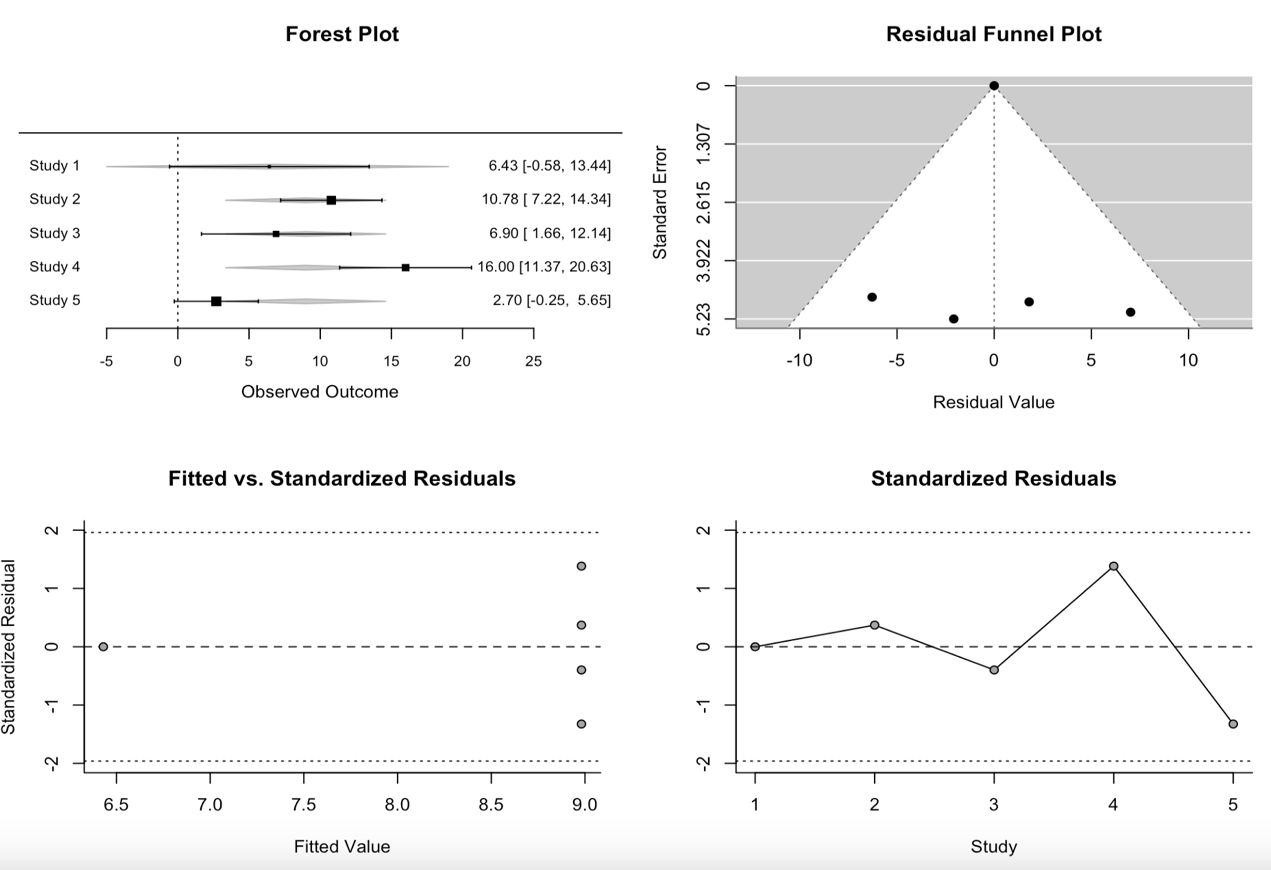


Figure S19 Figure of Meta regression analysis of Quality of Life


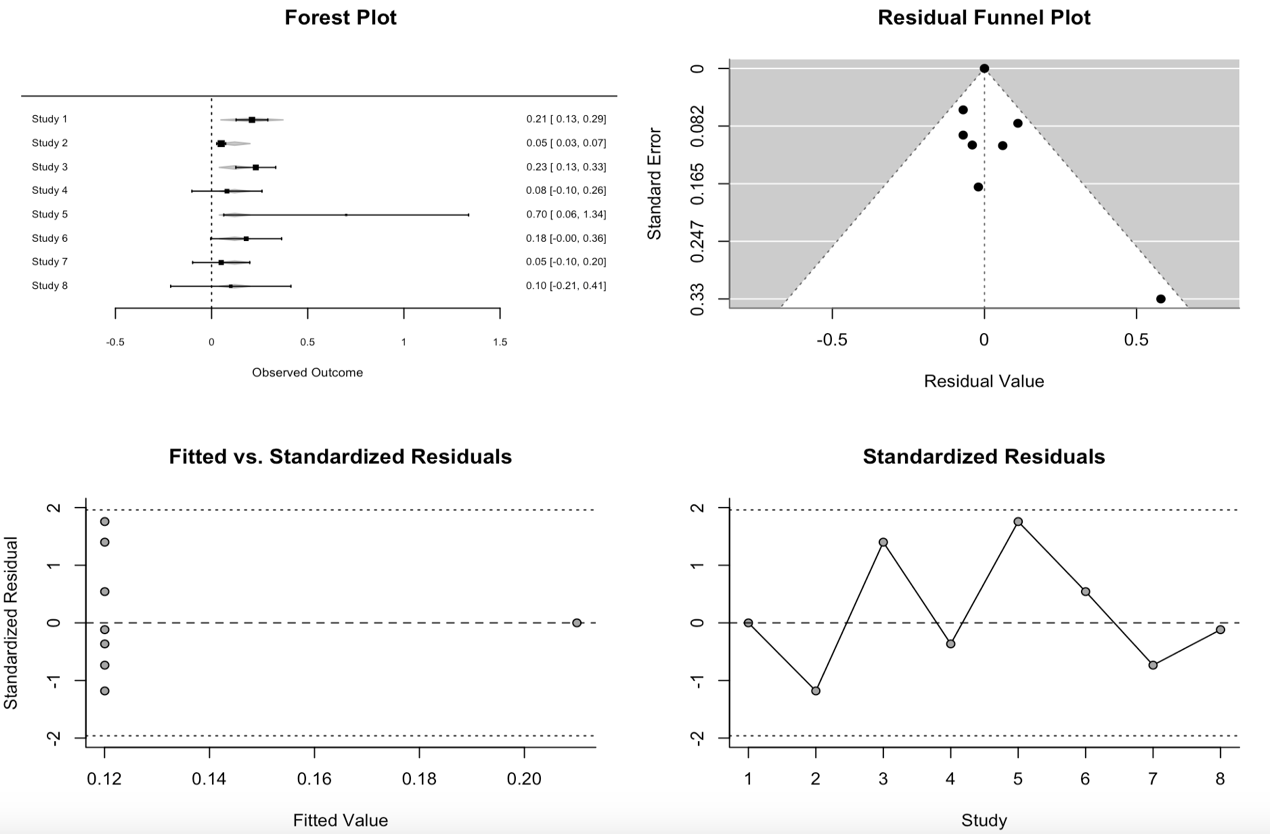


Figure S20 Figure of Meta regression analysis of FEV1


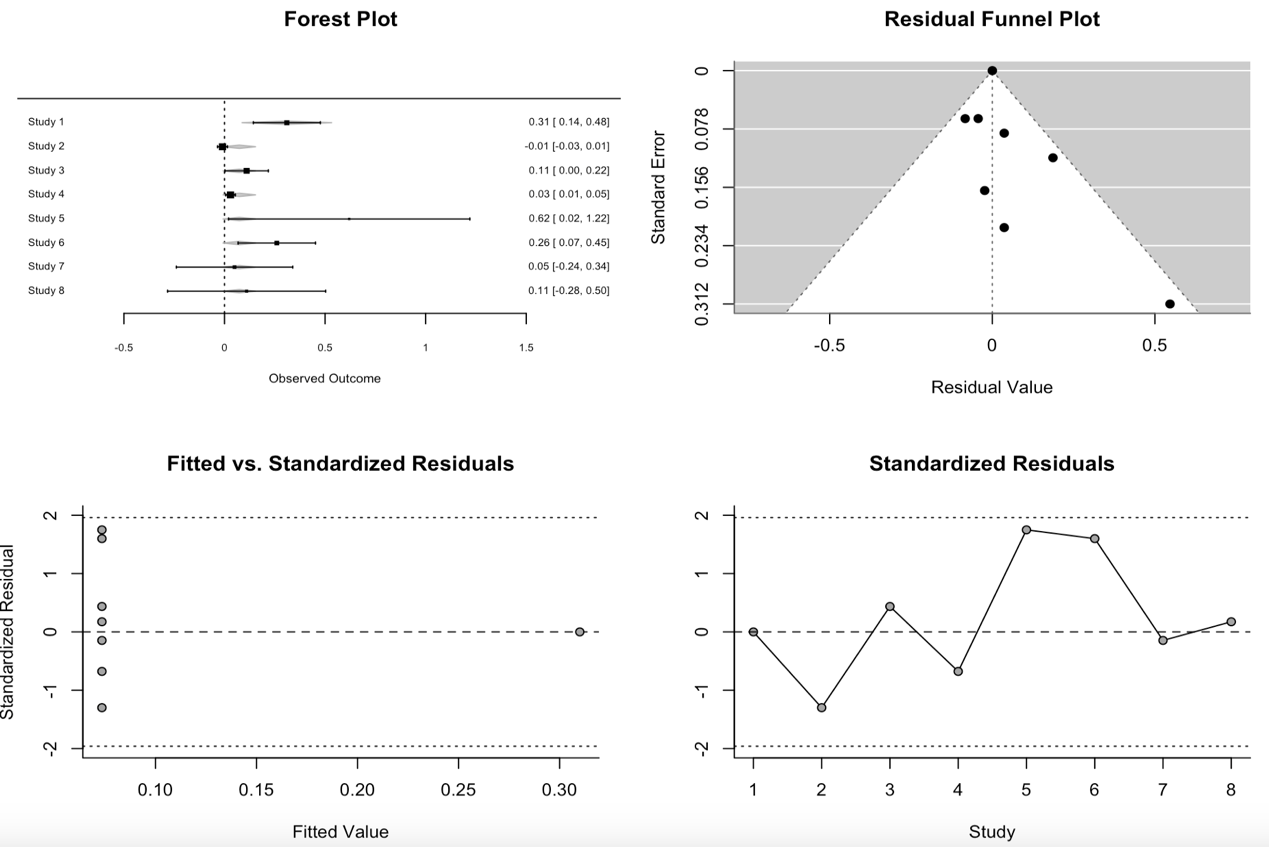


Figure S21 Figure of Meta regression analysis of FVC
